# Spatio‐Temporal Processes of Diffusion‐Controlled Communication in Hierarchical Multi‐Compartments

**DOI:** 10.1002/anie.202424133

**Published:** 2025-05-08

**Authors:** Xin Qiao, Haixu Chen, Andreas Schurig, Xiaoliang Wang, Yinyong Sun, Matthias Tobler, Susanne Boye, Kathrin Castiglione, Dietmar Appelhans, Xin Huang

**Affiliations:** ^1^ MIIT Key Laboratory of Critical Materials Technology for New Energy Conversion and Storage, School of Chemistry and Chemical Engineering Harbin Institute of Technology Harbin 150001 China; ^2^ Leibniz‐Institut für Polymerforschung Dresden e.V. Hohe Straße 6 01069 Dresden Germany; ^3^ Institute of Bioprocess Engineering Friedrich‐Alexander‐Universität Erlangen‐Nürnberg Paul‐Gordon‐Straße 3 91052 Erlangen Germany

**Keywords:** Azobenzene, Coacervates, Feedback‐controlled behavior, Spatio‐temporal processes, Phospholipid bilayer, Polymersomes

## Abstract

Exploring the synergy of feedback behavior and molecular communication between micro‐ and nanocompartments is of great implication for the development of advanced hierarchical living‐like materials. Non‐covalent interactions are the driving forces for dynamic and temporal events in biomimetic structures. Herein, pH‐responsive hierarchical multi‐compartments (HMC) are constructed via hydrophobic–hydrophobic interactions between azobenzene units and phospholipid layers through the integration of two distinct structural units: phospholipid‐membranized coacervates (Coa@DMPC) and azobenzene‐functionalized polymersomes (Azo‐Psomes). This enables us to study spatio‐temporal signal pathways for biomimetic pH homeostasis and the triggering of feedback‐controlled peroxidase‐like behavior of Azo‐Psomes within HMC. Compared with undocking systems, the information transmission process within HMC shows a high efficiency. Besides the continuous addition of nutrients, the synchronization of two different biomimetic reactions in HMC requires the spatial loading of glucose oxidase and L‐phenylalanine ammonia lyase in coacervates and of L‐phenylalanine or beta‐cyclodextrin/hemin complexes in Azo‐Psomes. Azo‐Psomes exhibit pH‐responsive feedback‐controlled behavior. The pH‐responsive membrane of Azo‐Psomes is responsible for the spatio‐temporal peroxidase‐like activity of lumen‐integrated beta‐cyclodextrin/hemin complexes in Azo‐Psomes. Finally, this strategy provides a new approach for constructing more complex biomimetic systems by interconnecting at least two membrane‐containing compartments to further explore the synergistic mechanisms and feedback behaviors among artificial cell communities.

## Graphical abstract

This study highlights the molecular communication of neighboring biomimetic populations in pH‐responsive hierarchically and non‐covalently organized multi‐compartments (HMC) through the assembly of micro‐ and nanocompartments. HMC unify key roles for cyclic spatio‐temporal diffusion processes and spatio‐temporal enzyme(‐like) reactions.

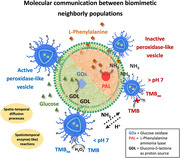

## Introduction

Organisms are made up of a multitude of cells, and they often form communities that combine their strengths to exhibit more advanced behaviors than those of individual cells. This allows them to continuously adapt to cellular changes in their environment and achieve more cell‐driven functions. This improves their micro‐/nano‐environmental survival.^[^ ^1^, ^2^ ^]^ Signal exchanges between different cellular communities are dynamic and can be generally attributed to fast adaptation to their environment. The release and detection of signal molecules in response to environmental changes will instantly verify their cell metabolism, thereby enhancing their cell capabilities in a changing micro‐ and nano‐environment. Researchers use artificial microcompartments and biologically active components with the objective of comprehensively understanding these intricate and dynamic collective behaviors. This approach involves mimicking analogous processes with the aim of elucidating the operational mechanisms of living systems.^[^ ^3^, ^4^, ^5^, ^6^, ^7^ ^]^

**Scheme 1:**
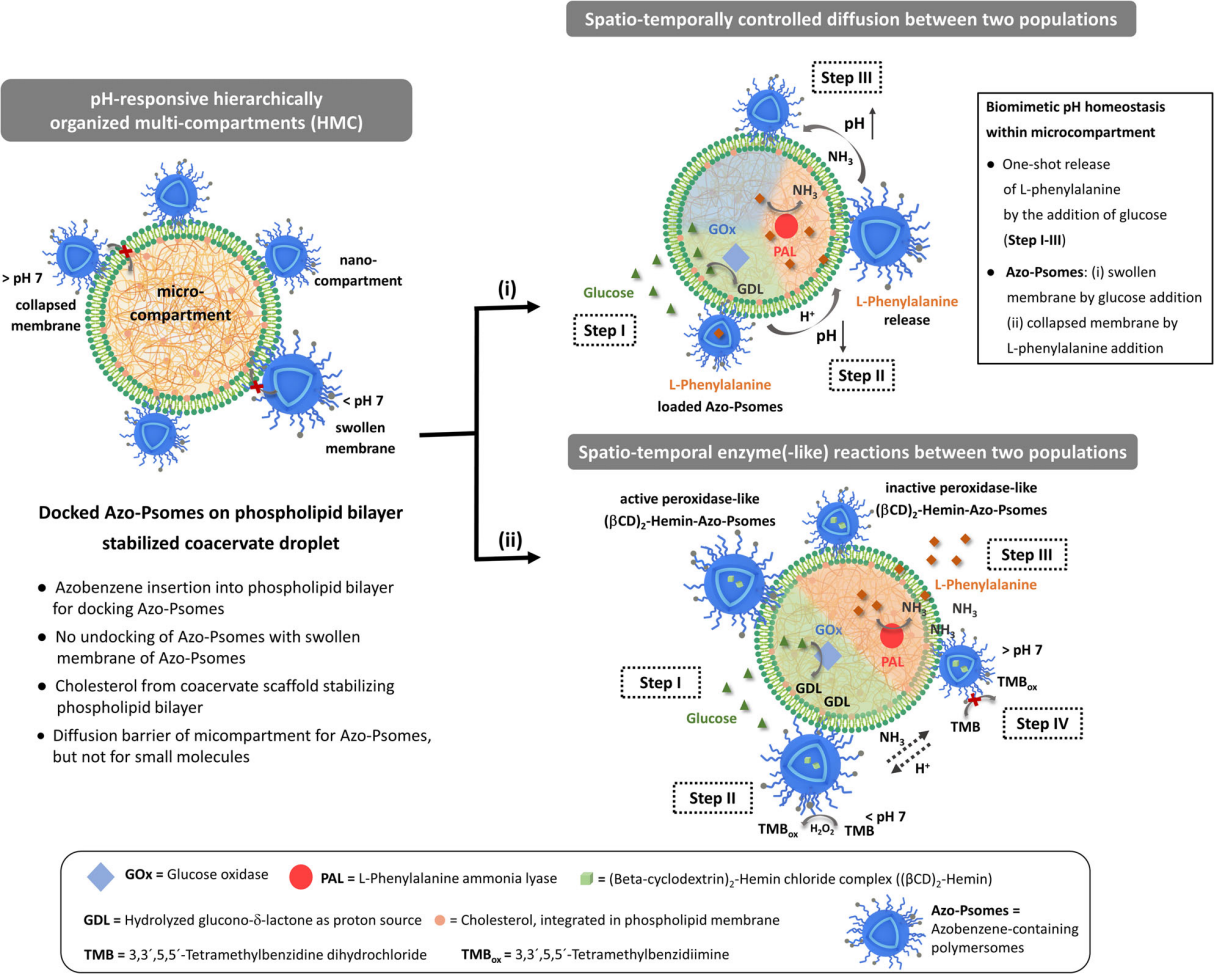
The formation of pH‐responsive HMC is accomplished by inserting azobenzene on Azo‐Psomes into the phospholipid bilayer on Coa@DMPC. Azo‐Psomes achieve swelling or shrinking on the Coa@DMPC surface without damaging the docking when pH changes. Based on the encapsulation capacity of Coa@DMPC and Azo‐Psomes to sequester biomacromolecules for carrying out (i) spatio‐temporal control of diffusion and (ii) spatio‐temporal enzyme(‐like) reactions through the use of HMC.

Currently, a wide range of microcompartments have been designed and constructed to simulate certain properties of living cells. These include liposomes,^[^ ^8^ ^]^ colloidosomes,^[^ ^9^ ^]^ polymersomes,^[^ ^10^, ^11^, ^12^, ^13^ ^]^ proteinosomes,^[^ ^14^, ^15^, ^16^ ^]^ and coacervates, among others.^[^ ^17^, ^18^, ^19^ ^]^ These artificial microcompartments are capable of communicating with each other. They exchange chemical and biological molecules with other cell communities, based on their characteristics.^[^ ^20^ ^]^ For example, inner compartments within liposomes allow for the continuous directional transfer of signaling molecules to facilitate enzymatic reactions within artificial cells.^[^ ^21^ ^]^ Feedback loops under light/dark cycles undoubtedly control environmentally responsive polymersomes.^[^ ^11^ ^]^ Additionally, the efficient transfer of chemical signals between heterologous artificial microcompartments is still demonstrated.^[^ ^22^, ^23^ ^]^ More importantly, bidirectional communication between artificial cells can be achieved through the diffusion of signal molecules^[^ ^24^ ^]^ and DNA strand displacement,^[^ ^25^ ^]^ demonstrating the dynamic information transmission process under feedback loops. Furthermore, the synergistic interaction between artificial cells is evident in the different transport modes achieved by the synergistic or antagonistic enzyme reactions of enzyme‐active semipermeable proteinosomes within a helical hydrogel filament.^[^ ^26^ ^]^ Environmental changes such as light,^[^ ^27^, ^28^ ^]^ temperature,^[^ ^29^ ^]^ and pH^[^ ^30^ ^]^ are useful chemical and physical stimuli that boost the communication process between artificial microcompartments and their environment. Among them, pH affects many biochemical processes in an organism and is therefore applied in artificial systems to mimic the dynamic behavior of living cells. Nevertheless, the current research focuses more on the unidirectional impact of the environment on the constructed artificial microcompartment community. However, there are few reports on the effective exchange of signaling molecules under synergistic interactions between two distinct artificial microcompartment communities.^[^ ^22^ ^]^ The synergistic interactions can be further strengthened through the feedback‐controlled behaviors of participating compartments in response to environmental (pH) changes. This suggests that spatio‐temporal diffusion processes are involved, whereby a controlled diffusion barrier must be overcome and, ultimately, dictates time‐dependent enzyme(‐like) processes. This principle is the focus of the recent study (Scheme 1).

To realize and investigate the postulated principles presented in our study (Scheme 1), the formation of HMC is established by the non‐covalent interactions between an excess nanocompartment, azobenzene‐containing polymersomes (Azo‐Psomes), and a minor microcompartment, phospholipid bilayer‐membranized coacervate (Coa@DMPC), where Azo‐Psomes undergo a surface decoration of Coa@DMPC. Coa@DMPC is attributed to the integration of cholesterol functional groups in the cationic polyelectrolyte (PMEDAB‐Chol). The cholesterol groups on the outer exterior of coacervate droplets facilitate the formation of a densely packed phospholipid bilayer coating which serves to stabilize the droplets. The azobenzene units present on the surface of Azo‐Psomes enable their insertion into the phospholipid bilayer that coats the surface of Coa@DMPC. This facilitates the rapid and efficient docking of the nanocompartment, Azo‐Psomes, to the microcompartment surface of Coa@DMPC, ultimately leading to the formation of the desired HMC.

The Coa@DMPC's capacity to sequester biomacromolecules is employed to achieve dynamic regulatory behavior between the nano‐ and microcompartment. This is achieved by encapsulating glucose oxidase (GOx) and L‐phenylalanine ammonia lyase (PAL). Through the use of HMC, spatio‐temporal control of diffusion processes and enzyme(‐like) reactions is carried out (Scheme 1). In the spatio‐temporally controlled diffusion processes (Scheme 1, i+ii), the addition of glucose triggers GOx to convert glucose into gluconic acid, lowering the pH inside and outside of HMC. This pH decrease induces the one‐time release of L‐phenylalanine from the pH‐responsive Azo‐Psomes. The released L‐phenylalanine is captured by coacervates, where PAL catalyzes ammonia production, thereby increasing the pH. HMC outperforms the undocking system, demonstrating high efficiency and energy savings with spatio‐temporal control of diffusion processes (Figure 5). In the case of spatio‐temporal enzyme(‐like) reactions within HMC (Scheme 1, ii), (βCD)_2_Hemin complexes, loaded within the Azo‐Psomes, function as peroxidase‐like enzymes in the lumen of Azo‐Psomes. Their activity is mainly controlled by the pH‐responsiveness of Azo‐Psomes, whose swollen or collapsed membrane state is responsible for active or inactive (βCD)_2_Hemin complexes, respectively (Figure 6). The Scheme 1 demonstrates the difference in enzyme‐like activity in response to a changing pH environment in HMC. Thus, this demonstrates that HMC can conjugate diverse biomimetic functions between the nano‐ and microcompartments, using permanent and spatio‐temporal permeable membranes for molecular communication. This finally results in dynamic self‐regulation characteristics that are not addressed in previous bidirectional communication systems,^[^ ^24^, ^25^, ^26^ ^]^ thereby establishing the foundation for constructing more complex biomimetic systems in the future.

## Results and Discussion

### Establishing Phospholipid Bilayer Membranized Coacervates and Azobenzene‐Containing Polymersomes for the Construction of pH‐Responsive Hierarchical Multi‐Compartments

**Figure 1:**
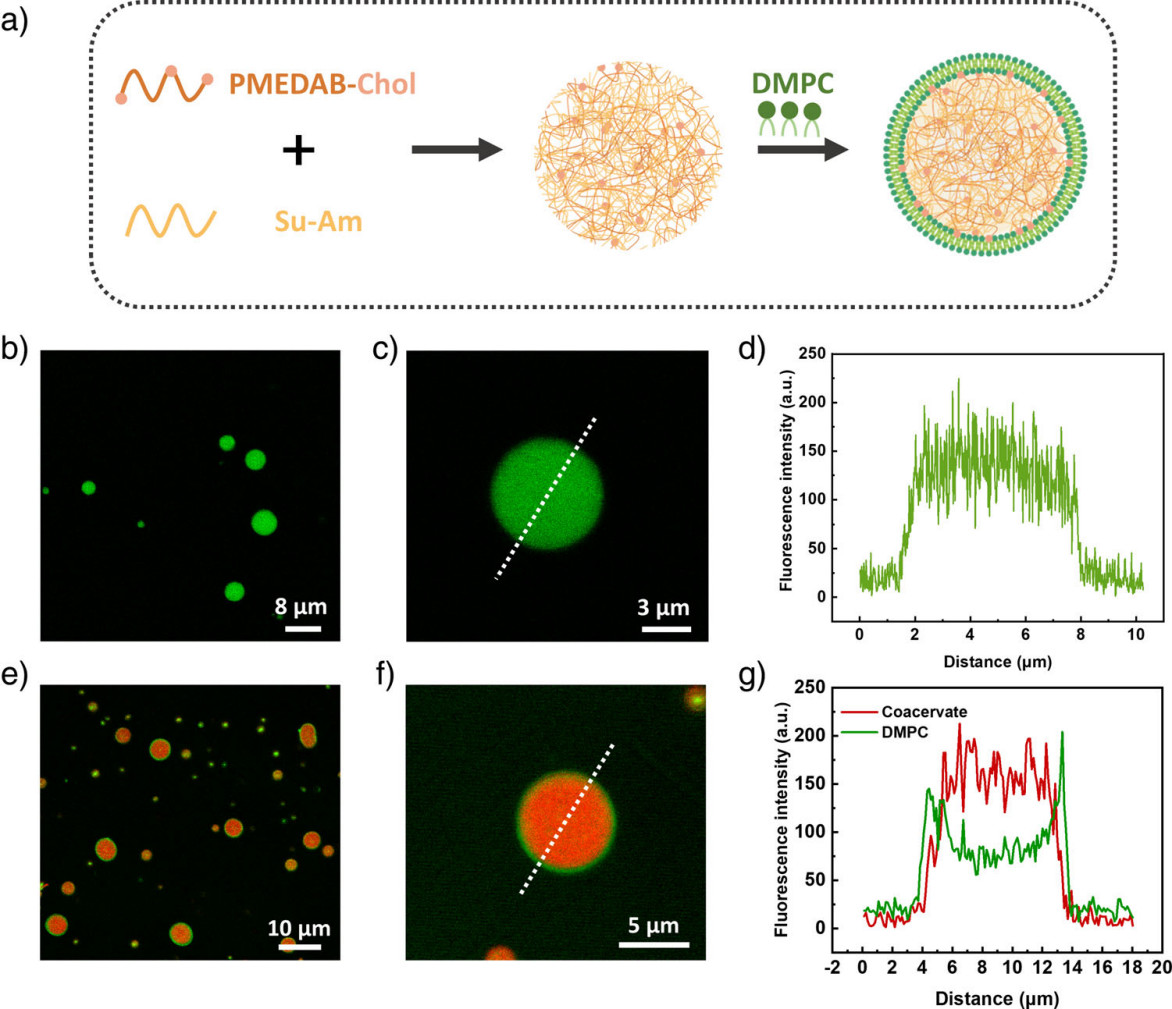
a) Schematic illustration showing the fabrication process of Coa@DMPC. Coacervate microdroplets formed by the hydrophobic and electrostatic interactions between Su‐Am and PMEDAB‐Chol (the concentration ratio of PMEDAB‐Chol and Su‐Am is 2:1), followed by the addition of DMPC in the coacervate solution, where phospholipids enriched around the surface of coacervate droplets. b) Fluorescein‐loaded coacervate droplets, and c),d) corresponding fluorescence intensity analysis (position of the imaginary line is shown in c). e) CLSM images showing the DMPC (NBD‐PC, green)‐based phospholipid membrane around the coacervates (RhB, red). f), g) Cross‐section analysis of single coacervate in (e), showing a preferred location of DMPC around the outer surface of Coa@DMPC.

The concept of cholesterol anchoring to and stabilization of phospholipid membranes^[^ ^31^, ^32^, ^33^ ^]^ is followed in phospholipid membranized coacervate droplets (Coa@DMPC) (Figure 1a). Coacervate droplets are fabricated by the preferential electrostatic interaction of cholesterol‐modified cationic polyelectrolyte (PMEDAB‐Chol; Figures S3–S5) with the anion succinylated amylose (Su‐Am; Figure S2), finally characterized by confocal laser scanning microscopy (CLSM) and zeta potential measurements (Figures 1b–d, S7).

The Supporting Information (SI) presents the synthesis and characterization of both polyelectrolytes. Figure 1e–g highlights the successful phospholipid membranization of coacervates after the addition of 1,2‐dimyristoyl‐syn‐glycero‐3‐phosphocholine (DMPC). Due to the presence of hydrophobic cholesterol in coacervate, a lower part of phospholipids is also integrated in the coacervate phase due to the anchoring effect of cholesterol. Briefly, the membrane‐enriched DMPC layer (green fluorescence of DMPC by NBD‐PC) on the coacervate surface (red fluorescence by RBITC) is confirmed by CLSM images. The presence of phospholipids on the surface of the coacervates is clearly visualized by cross‐section and overlay analysis of the CLSM image, indicating that the phospholipid, DMPC, is preferentially located at the outer surface of coacervate. When β‐cyclodextrin is used to replace the molecular interactions between cholesterol and phospholipids,^[^ ^34^, ^35^ ^]^ the cholesterol‐stabilized phospholipid membrane of Coa@DMPC is immediately weakened and disappears within seconds. The fluorescent NBD‐PC for the visualization of DMPC‐containing phospholipid membrane completely diminishes and also disappears in the coacervate matrix. This proves that the molecular interaction between cholesterol and phospholipids is one of the key driving forces for the assembly and stabilization of phospholipid membranes on the surface of the coacervate (Figure S6). Results of zeta potential measurements outline that the Coa@DMPC surface provides a slightly positive surface charge (≤10 mV), as similarly found for the coacervate droplets (Figure S7). This indicates that the zwitterionic phospholipid membrane has no significant influence on the original surface charge of the coacervate droplets.

**Figure 2:**
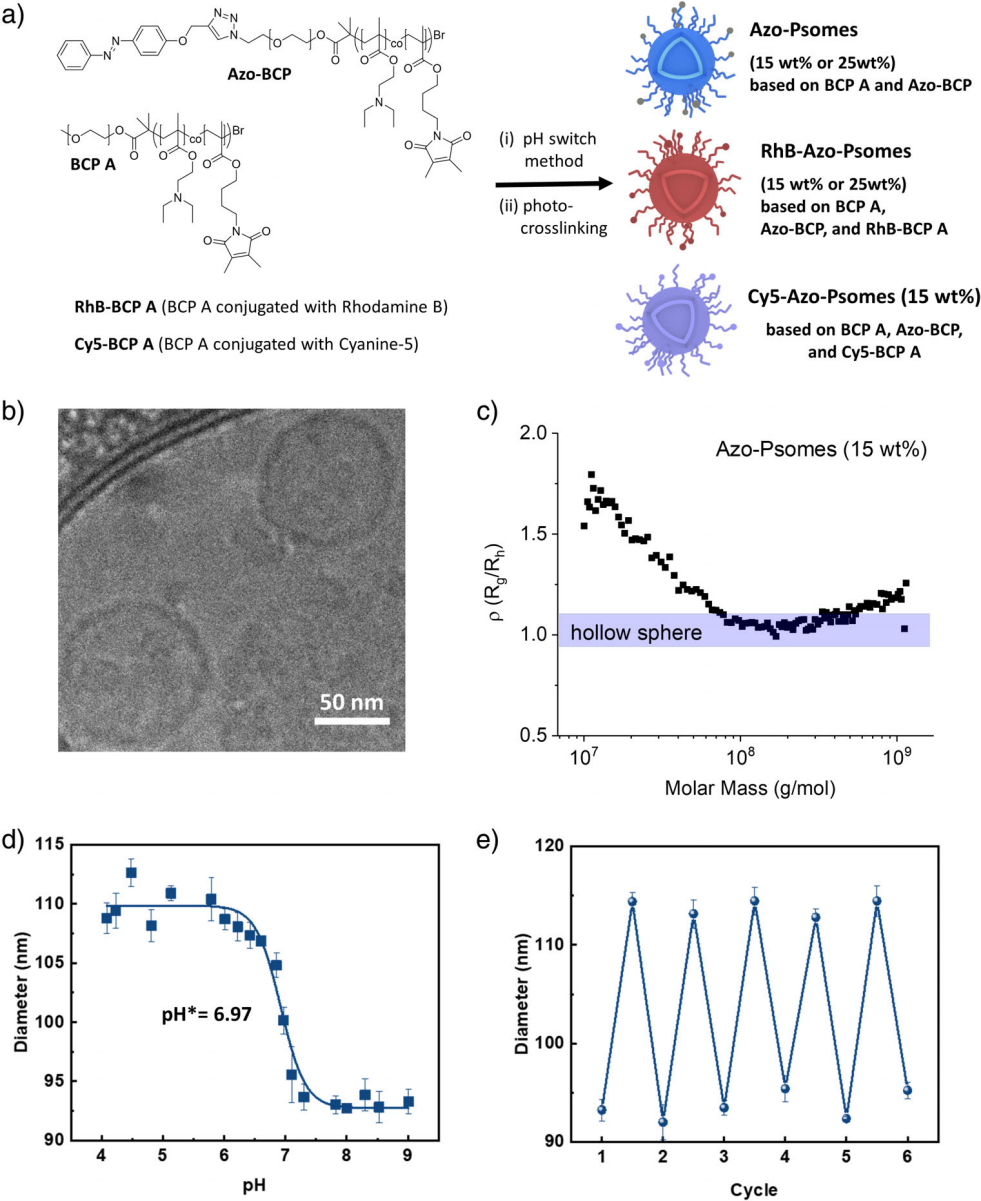
a) Fabrication procedure for (dye‐labeled) Azo‐Psomes (15 or 25 wt%); chemical structure and synthesis of BCP and Azo‐Psomes are presented in the SI. b),c) Characterization of Azo‐Psomes (15 wt%) by cryo‐TEM and asymmetric flow‐field flow fractionation conjugated with light scattering (AF4‐LS); rho (ρ = R_g_/R_h_) parameter as a function of molar mass determined by AF4‐LS; further details of AF4‐LS discussion at Figure S30. d) pH‐dependent size change of Azo‐Psomes (15 wt%) by pH‐dependent DLS measurements. e) The size change of Azo‐Psomes (15 wt%) during cyclic pH‐switches of Azo‐Psomes (15 wt%) between pH 4 and 8 determined by DLS. (*n* = 3, mean ± SD).

**Figure 3:**
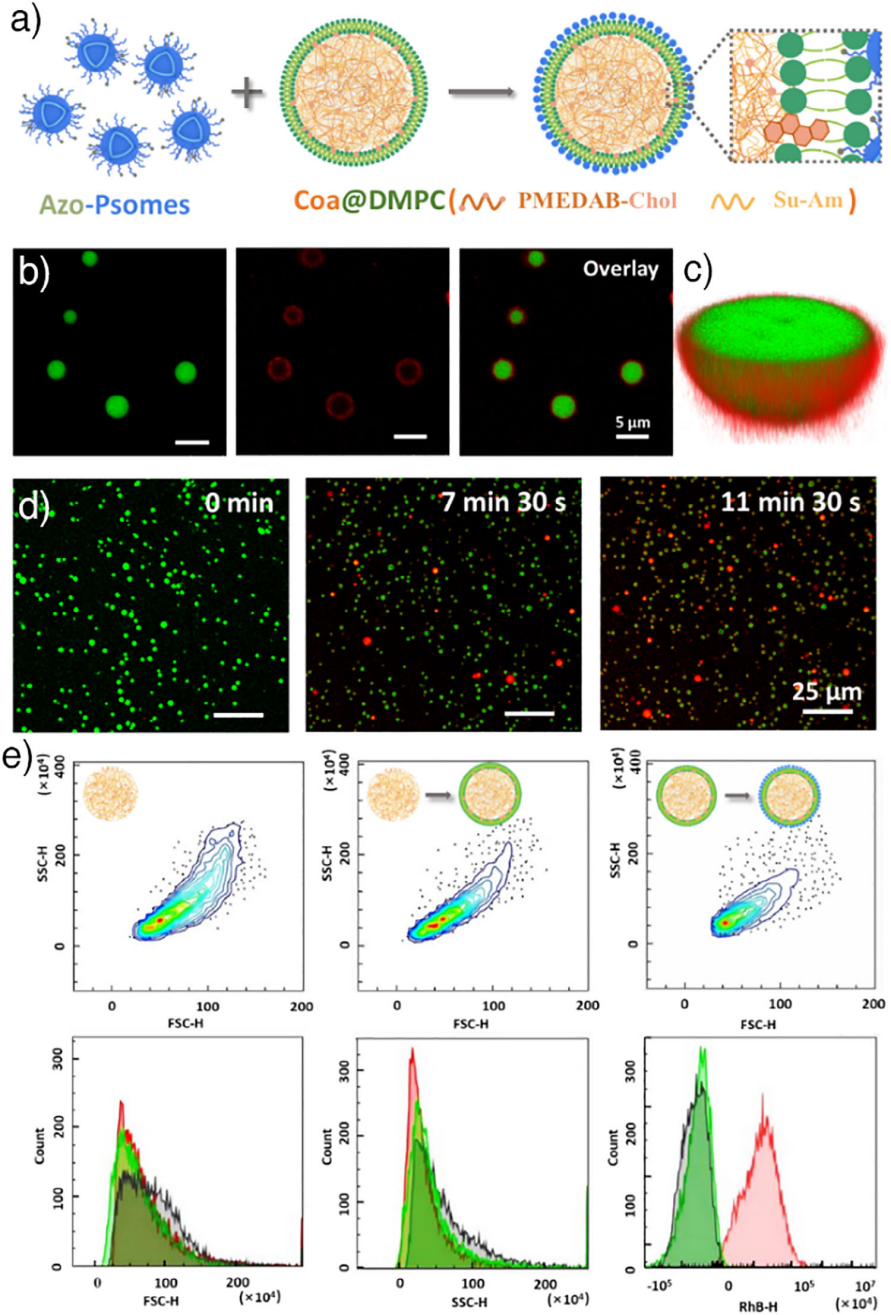
a) Schematic illustration showing that the azobenzene surface groups of Azo‐Psomes are able to insert into the phospholipid layer on the surface of the coacervate, leading to the docking of the nanocompartment to the surface of the microcompartment. b), c) CLSM images indicating the RhB‐Azo‐Psomes (red) docking to the surface of FITC‐labeled Coa@DMPC (green) via the fluorescence colocalization analysis and 3D confocal image in (c) (The volume ratio of Azo‐Psomes (15 wt%) to Coa@DMPC is 2:1). d) Time‐dependent corresponding fluorescence microscopy images of RhB‐Azo‐Psomes (red) around FITC‐labeled Coa@DMPC (green) (The volume ratio of Azo‐Psomes (15 wt%) to Coa@DMPC is 2:1). e) FACS‐derived 2D contour maps of side‐scattered light (SSC) versus forward‐scattered light (FSC) for coacervates, Coa@DMPC, and binary population after mixing Coa@DMPC and Azo‐Psomes, and corresponding plots of counts against FSC‐H values, SSC‐H values, and the red fluorescence signal (RhB‐H). The grey area is the coacervate droplet, the green area is Coa@DMPC, and the red areas are binary populations after mixing Coa@DMPC and Azo‐Psomes.

To achieve the final pH‐responsive HMC (Figure 3) by the docking of Azo‐Psomes onto Coa@DMPC surfaces, pH‐responsive Azo‐Psomes are constructed according to the reported method through the co‐assembly of pH‐responsive BCP A (Figure S8, Scheme 1) as main component and Azo‐BCP (Figure S9, Scheme 1) as minor component, using a pH‐switch method (Figure 2a).^[^ ^29^ ^]^ The synthesis and characterization of amphiphilic BCP A and Azo‐BCP, as well as Azo‐Psomes (15 and 25 wt%), are presented in the SI. In the final control experiments for the preparation of HMC, Azo‐Psomes (25 wt%) with a higher content of azobenzene units as surface groups are also used to confirm the docking potential of Azo‐Psomes on Coa@DMPC. In this work, Azo‐Psomes (15 wt%) are selected as the docking nanocompartment without special annotation in this study. The vesicular structure of Azo‐Psomes is verified by cryogenic‐transmission electron microscopy (cryo‐TEM) (Figure 2b) and conformation analysis AF4‐LS (Figures 2c, S30). Azo‐Psomes (15 wt%) have values of ρ parameters around 1 for the main fraction (Figure 2c), which is characteristic of hollow spheres^[^ ^36^ ^]^ in the range of 10^8^–10^9^ g mol^−1^. This observation corresponds to previously published data for Azo‐Psomes (25 and 50 wt%) with vesicular shapes (further details below Figure S30).^[^ ^29^ ^]^ Image of cyro‐TEM outlines a typical vesicular structure in the expected diameter range (Figure 2b).

Figure 2d,e clearly illustrates the expected pH‐responsiveness and structural robustness of Azo‐Psomes, as investigated by dynamic light scattering (DLS). The critical pH value (pH*) represents the half‐power swelling or the turning point between swelling and shrinking actions, revealing a pH* of 6.97 for Azo‐Psomes. Azo‐Psomes undergo the multiple acid‐base reciprocal changes at pH 8 (shrunken state = collapsed membrane state) and 4.0 (swollen membrane state) for 5 cycles. The pH‐responsiveness of Azo‐Psomes is similar to previously published results of pH‐responsive (Azo‐)Psomes.^[^ ^10^, ^12^, ^29^ ^]^ Notably, Rhodamine B (RhB) labeling of Azo‐Psomes with integrated RhB‐labeled BCP A (Scheme 1, Figure S10) does not alter their pH‐responsive properties (Figure S11), at which RhB units are integrated in the membrane of Azo‐Psomes and cannot manipulate the insertion properties of azobenzene units as surface groups in Azo‐Psomes toward Coa@DMPC during the docking process, validated by CLSM (results are presented below).

### Establishing pH‐Responsive Hierarchical Multi‐Compartments for Spatio‐Temporal Control of Diffusion Processes and Reactions

As demonstrated in previous studies, the hydrophobic structure of azobenzene can insert into phospholipid bilayers.^[^ ^37^, ^38^, ^39^ ^]^ Similarly, the azobenzene functional groups on the surface of Azo‐Psomes can also efficiently insert into the phospholipid layer of the Coa@DMPC to realize the directional docking of Azo‐Psomes to the microcompartments, Coa@DMPC (Figure 3a). CLSM images (Figure 3b–d) show the following results that RhB‐Azo‐Psomes (15 wt%, red color) discontinuously and time‐dependently dock on the surface of FITC‐labeled Coa@DMPC (green color) after some min. Alternatively, the use of RhB‐Azo‐Psomes (25 wt%) resultin a denser docking to the surface of FITC‐labeled Coa@DMPC (green color) becomes denser (Figure S12). The enhanced azobenzene content increases the docking probability with the phospholipid bilayer by increasing molecular interactions. Furthermore, systematic optimization of Azo‐Psome/Coa@DMPC volume ratios (1:1, 2:1, 10:1) reveals that Azo‐Psomes (15 wt%) achieve progressively higher docking densities on the microcompartments at higher ratios, demonstrating ratio‐dependent interfacial assembly behavior (Figures S13 and S14).

To verify that the docking of Azo‐Psomes to Coa@DMPC surface is due to the interaction of azobenzene with the phospholipid bilayer, a series of controlled experiments are performed. The experiments confirm that the docking of Azo‐Psomes to Coa@DMPC surface is due to the interaction of azobenzene with the phospholipid bilayer. Azo‐Psomes have a small size range of 90–120 nm (Figure 2d,e). Both RhB‐Azo‐Psomes (15 wt% and 25 wt%) they are too small to be directly observed by CLSM (Figures S15 and S16). Only after docking to the Coa@DMPC surface the obvious red fluorescence of both Azo‐Psomes can be observed (Figures S13 and S14). In contrast, when the coacervate without DMPC membranization is incubated with RhB‐Azo‐Psomes (25 wt%) for a long period (30 min), no docking is observed (Figure S17). Carrying out a similar experiment with Coa@DMPC and Psomes A without azobenzene (Details on the fabrication of Psomes A in the SI), no docking is observed on the zwitterionic surface of Coa@DMPC (Figure S18). In conclusion, the docking mechanism of Azo‐Psomes to the microcompartment, Coa@DMPC, is based on the insertion of azobenzene into the phospholipid bilayer. In short, this also indicates that the combination of PEG shell and azobenzene surface groups of slightly cationic Azo‐Psomes are not able to undergo any non‐covalent interactions with the cationic coacervate droplets.^[^ ^16^, ^29^ ^]^ In the case of the pure PEG shell of Psomes A, the undocking or unattachment of Psomes A is explainable due to the protein‐repellent properties of the PEG shell and zwitterionic surface of Coa@DMPC.

To further prove the successful docking of Azo‐Psomes to Coa@DMPC surface, fluorescence activated cell sorting (FACS) analysis is also performed to validate coacervate populations before and after the membranization of DMPC (Figure 3e). The results show similar side‐scattered (SSC) signals, while forward‐scattered (FSC) signals decrease slightly. The latter observation is attributed to the stabilization and homogenization of the coacervate size after DMPC based membranization.

When RhB‐Azo‐Psomes are further bound to Coa@DMPC, neither SSC nor FSC show a significant change. This is caused by the fact that the small size of RhB‐Azo‐Psomes does not alter the size and complexity of Coa@DMPC upon docking. However, the related average fluorescence measurements show that the red fluorescence intensity of RhB‐Azo‐Psomes is also visible on Coa@DMPC surfaces after the docking process, intuitively confirming the occurrence of the docking between the Azo‐Psomes and Coa@DMPC populations (Figure 3e).

### Requirements of pH‐Responsive Hierarchical Multi‐Compartments for Functioning Spatio‐Temporal Control of Diffusion Processes and Reactions

**Figure 4:**
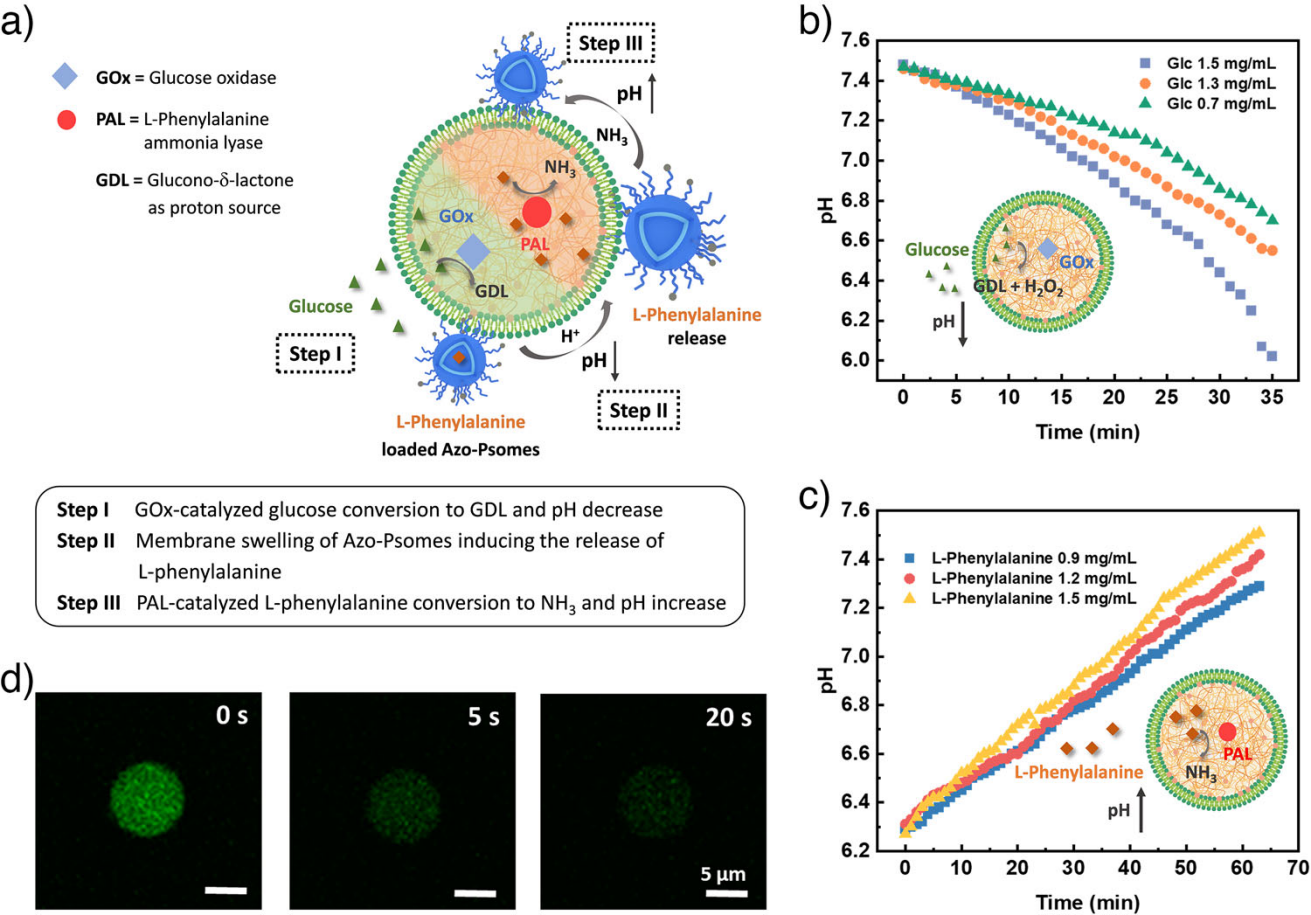
a) Schematic representation of the pH‐responsive hierarchical multi‐compartments for functioning spatio‐controlled diffusion processes and reactions. Carrying out control experiments b)–d) for functioning HMC to undergo one‐time release of L‐phenylalanine from Azo‐Psomes (steps I‐III). GOx‐converted glucose into gluconic acid, lowering the pH of the environment. This pH drop triggers the release of L‐phenylalanine from Azo‐Psomes, which is then captured by coacervate matrix. PAL catalyzes ammonia production to increase the pH. b) pH changes of Coa@DMPC loaded with GOx in PBS solution at pH 7.5 after adding different concentrations of glucose. c) pH changes of Coa@DMPC loaded with PAL in PBS solution at pH 6.3 after adding different concentrations of L‐phenylalanine. d) The in situ fluorescence changes of FITC in Coa@DMPC loaded with GOx after adding glucose (See Figure S20 for details).

The successful docking of Azo‐Psomes to the surface of Coa@DMPC is a prerequisite to further establish a bridge between the two populations and for the associated feedback behaviors (Figure 4a). To demonstrate the final functions of spatio‐temporal control of diffusion processes and enzyme(‐like) behaviors in HMC (Scheme 1), the Coa@DMPC should be loaded with GOx and PAL, and Azo‐Psomes should be loaded with L‐phenylalanine. Then with the addition of glucose, gluconic acid will be produced catalyzed by GOx inside Coa@DMPC. This should lead to the decrease of pH and, finally, to the swelling of Azo‐Psomes, accompanied by the one‐time release of L‐phenylalanine. Thus, the released L‐phenylalanine should be preferentially sequestered by the Coa@DMPC compartment of the pH‐responsive HMC and then be converted by PAL to release ammonia inside and outside the HMC. The final expectation should be the increase of pH, resulting in the feedback‐controlled contraction of Azo‐Psomes to regain an impermeable Azo‐Psomes membrane.

**Figure 5:**
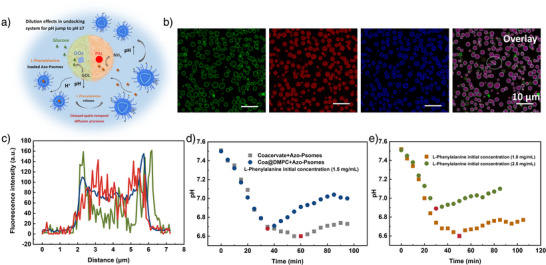
Determination of the molecular composition of HMC b), c) and biomimetic pH homeostasis d), e), only presenting the one‐time release of L‐phenylalanine from HMC with L‐phenylalanine‐loaded Azo‐Psomes (d). a) Schematic representation of the undocking system for delayed spatio‐temporal diffusion processes. b) CLSM images of HMC (Scheme 1), showing the DMPC (NBD‐PC, green) around the Coa@DMPC, loaded with PAL (Cy7, red) and GOx (Cy5, blue). Azo‐Psomes (Cy5, blue) show stronger blue fluorescence than the interior of GOx (Cy5, blue) after docking to the surface of Coa@DMPC. c) Corresponding fluorescence intensity analysis of HMC composition (position of the imaginary line is shown in (b)). d) Potential of one‐time release of L‐phenylalanine within HMC compared with the undocking system: (i) pH changes of HMC (Azo‐Psomes loaded with L‐phenylalanine docked onto the surface of Coa@DMPC, loaded with PAL and GOx) after adding glucose (1.5 mg mL^−1^); and (ii) Azo‐Psomes loaded with L‐phenylalanine in the presence of coacervate droplets, loaded with GOx and PAL, after adding glucose (1.5 mg mL^−1^). e) Potential of different initial concentrations of L‐phenylalanine for one‐time release within HMC: (i) L‐phenylalanine (1 mg mL^−1^), glucose (1.5 mg mL^−1^); and (ii) L‐phenylalanine (2.5 mg mL^−1^), glucose (1.5 mg mL^−1^).

**Figure 6:**
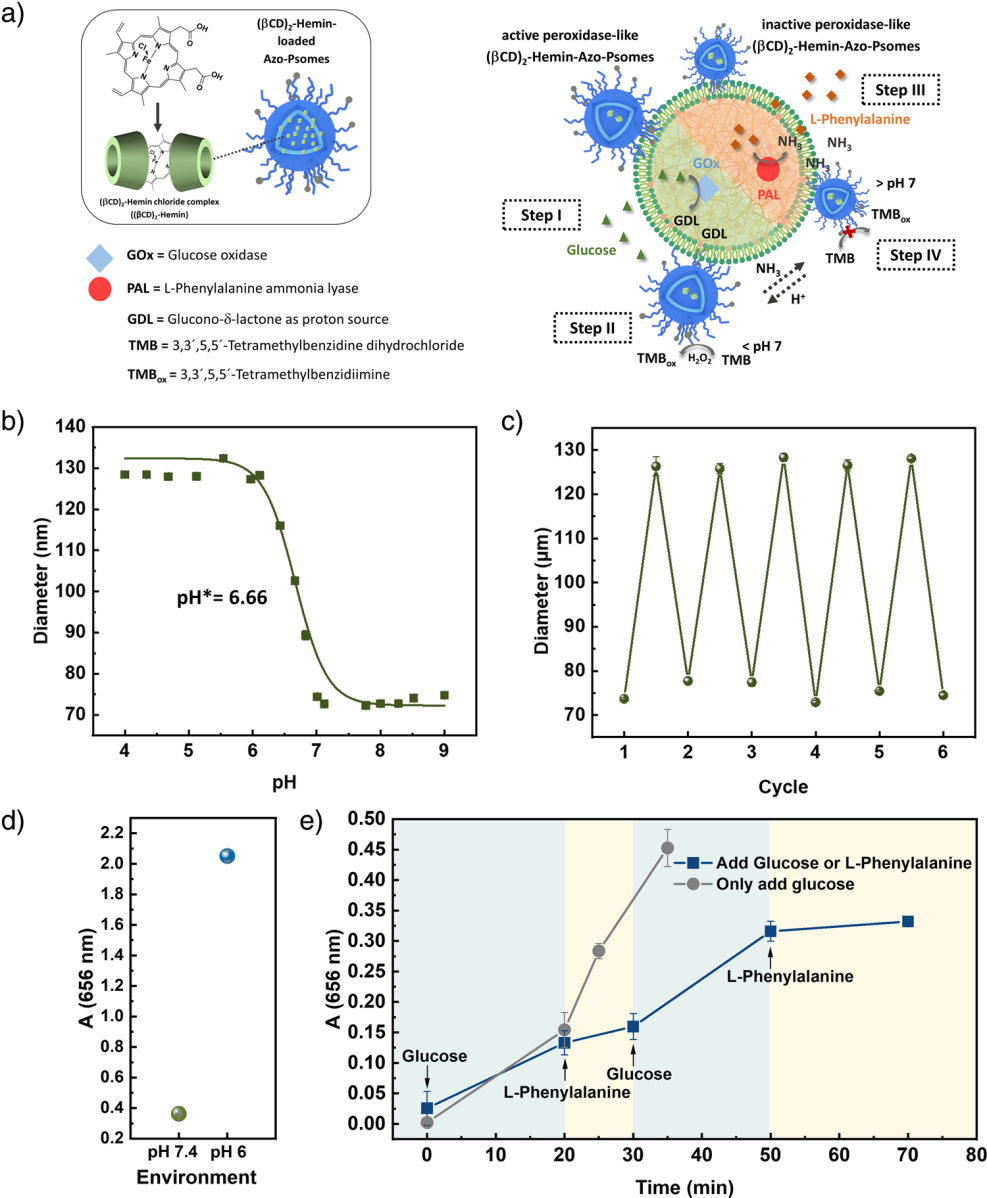
a) Schematic presentation of spatio‐temporal enzyme(‐like) reactions in HMC, using Azo‐Psomes loaded with (βCD)_2_Hemin to check biomimetic pH homeostasis‐dependent peroxidase‐like behavior. b) pH‐dependent size change of (βCD)_2_Hemin‐Azo‐Psomes (15 wt%) determined by DLS. c) Cyclic pH‐switches of (βCD)_2_Hemin‐Azo‐Psomes (15 wt%) between pH 4 and pH 8 determined by DLS. d) TMB assay of purified (βCD)_2_Hemin‐Azo‐Psomes (15 wt%) under different environments by UV–vis spectrometry at pH 7.4 and 6. e) Validation of peroxidase‐like activity for HMC (a) by TMB assay, using purified (βCD)_2_Hemin‐Azo‐Psomes (15 wt%) and Coa@DMPC, loaded with GOx and PAL. pH was changed by (i) sequential addition of glucose and L‐phenylalanine and (ii) time‐dependent addition of glucose only. (*n* = 3, mean ± SD).

The final experiments (Figures 5 and 6) involve checking specific requirements. During the formation of Coa@DMPC, enzymes GOx and PAL can be encapsulated separately to induce pH drops and jumps, respectively (Figure 4b,c). GOx‐loaded Coa@DMPC adjusts the environmental pH in response to varying glucose concentrations (Figure 4b). This demonstrates that small molecules, like glucose, can traverse the phospholipid bilayer of HMC (Figure 4a) and be oxidized by GOx into gluconic acid, releasing protons to lower the pH to 6 within 35 min at the highest glucose concentration (1.5 mg mL^−1^). Recombinantly expressed and purified PAL (details in SI) exhibits specific activity at different pH values (Figure S19), facilitating pH regulation in Coa@DMPC (Figure 4c) and HMC (Figures 5 and 6). The rate of pH increase and its upper limit can be modulated by L‐phenylalanine concentration.

Concluding the first required experiment series to follow pH drops and jumps by pH meter, pH changes in Coa@DMPC solution are slow and delayed due to dilution effects under the given experimental conditions. In order to more visually detect the instantaneous pH change of Coa@DMPC loaded with GOx upon the addition of glucose, an integrated pH‐sensitive dye, fluorescein, in GOx‐loaded Coa@DMPC can undoubtedly be used as an optical pH sensor for individual Coa@DMPC droplets due to the decreasing fluorescence properties of fluorescein at decreasing pH values. Figure 4d presents the strength of fluorescein as a pH sensor after the addition of a high glucose concentration (1.5 mg mL^−1^). Within 20 s fluorescence emission of fluorescein is preferentially quenched and finally corresponds to a pH value of 5.2. The results of a control experiment shown in Figure S20 further confirm the observation, presented in Figure 4d. Moreover, the observed pH jumps and drops, initiated by GOx and PAL catalyzed conversion steps (Figure 4a–c), trigger the feedback‐controlled swelling and deswelling of Azo‐Psomes (Figure 2d,e), finally supporting the establishment of requirements of spatio‐temporal diffusion processes and enzyme(‐like) reactions (Scheme 1).

After verifying the encapsulation ability of Coa@DMPC and the accompanied enzyme reaction‐triggered pH changes (Figure 4), it is necessary to demonstrate the pH‐stability of docked Azo‐Psomes in HMC at acidic pH (Figure S21). The docking of Azo‐Psomes to Coa@DMPC surface in HMC is not affected by the change in pH over 55 h while the pH is maintained around pH 6.5 (Figure S21; further details indicated). This implies that swollen Azo‐Psomes do not undergo preferential undocking processes from Coa@DMPC surface. Thus, HMC in acidic environment are stable and smoothly compensate the cationic repulsion forces of cationic Azo‐Psomes^[^ ^40^ ^]^ and cationic Coa@DMPC (Figure S7) by the preferentially existing hydrophobic‐π interactions through the insertion of azobenzene units into the phospholipid bilayer. This pH‐stable docking characteristic of Azo‐Psomes within HMC provides a further prerequisite for the following study of the feedback‐ and spatio‐temporally controlled diffusion and enzyme(‐like) processes (Figures 5 and 6). Finally, CLSM images clearly indicate the simultaneous desired encapsulation of enzymes, Cy5‐labeled GOx and Cy7‐labeled PAL, in Coa@DMPC, NBD‐labeled DMPC (NPC‐PC) (Figure S22, including cross‐section analysis) and the subsequent docking of Azo‐Psomes (Cy5‐labeled Azo‐Psomes; using Cy5‐labeled BCP for Azo‐Psomes formation; Scheme S2, Figure S23) onto the Coa@DMPC surface (Figure 5b,c) for the final construction of HMC (Scheme 1). It can be clearly seen that Cy5‐labeled GOx and Cy7‐labeled PAL enzymes are mainly loaded in Coa@DMPC, and the docking of Azo‐Psomes does not affect the loading of enzymes (Figure 5b). Although the fluorescence of Cy5‐labeled GOx is the same as that of Cy5‐labeled Azo‐Psomes (Figure 5b,c), the analysis of the integrated fluorescence intensity as well as the direct observation illustrates a stronger blue fluorescence on the membrane (blue cross‐section in Figure 5c). This confirms the docking of Azo‐Psomes to the Coa@DMPC surface, which also correlates with the afore‐mentioned results in Figure 3.

### Comparing the Cycle Potential of pH Drop and Jump by Enzyme‐Loaded HMC and the Undocking System, Containing Azo‐Psomes and Enzyme‐Loaded Coacervates

To demonstrate the potential of the enzyme‐loaded pH‐responsive HMC (Figure 4a) for spatio‐temporally controlled diffusion processes and regulated enzymatic reactions via biomimetic cyclic pH switches, a undocking system, consisting of L‐phenylalanine‐loaded Azo‐Psomes (Figures S24 and S25) and GOx‐/PAL‐loaded coacervates, is validated for a one‐time pH drop and jump. This system is then compared to the established enzyme‐loaded pH‐responsive HMC (Figure 5d, Scheme 1). Both systems demonstrate different concentration‐dependent one‐time releases of L‐phenylalanine from Azo‐Psomes.

The undocking system outlines a similar one‐shot release of L‐phenylalanine, but with a simultaneous initiation and slightly stronger pH drop (≤pH 6.6) compared to the pH‐responsive HMC (Figure 5a,d). In contrast, the slightly stronger pH drop of the undocking system is not helpful for a rapid unidirectional transfer of L‐phenylalanine or a controlled diffusion process from released L‐phenylalanine by Azo‐Psomes to non‐membranized and PAL‐loaded coacervate to produce ammonia needed to induce the requested pH jump to a pH of ≥pH 7. pH values of at least 7 are needed to induce the requested feedback‐controlled deswelling of Azo‐Psomes (Figure 2e) into the impermeable state of Azo‐Psomes membrane for the cyclic enzyme‐induced pH‐switches (Figure S28) and to tune the spatio‐temporal enzyme‐like reactions in the final series of experiments (Figure 6). Higher concentration on L‐phenylalanine‐loaded Psomes in the undocking system (Figure S26) induces a similar pH response behavior to that of MHC (Figure 5d). In addition, membrane‐less coacervates in the undocking system are easy to coalesce and dissociate, which will fail to establish stable HMC (Figure S27). For an effective biomimetic system, it is important to develop a stable low‐concentration, functioning HMC system with spatio‐temporally controlled diffusion processes.

In contrast, the docking Azo‐Psomes to the Coa@DMPC surface in HMC reduces the distance between the populations. This enhances the transfer of nutrient (glucose and L‐phenlalanine) and metabolite (gluconic acid and ammonia) within HMC. Moreover, the enzymatic reaction rate and kinetics of this process are regulated by the substrate concentrations. When the amount of L‐phenylalanine in Azo‐Psomes is too low, the pH jump is difficult to achieve, while the reaction rate and reaction time are effectively increased when the amount of L‐phenylalanine is increased (Figure 5e).

In summary, the constructed enzymatic reactions in HMC (Figures 5, S28) take place efficiently, triggered by the proximity of the two populations. This is not the case in the unstable non‐docking system at initially lower nutrient concentrations. Thus, HMC outlines the required feedback‐controlled characteristics (Figure S28) for the final study on spatio‐temporal enzyme(‐like) reactions (Scheme 1). This also demonstrates the potential of intramolecularly‐driven compartmental communication in HMC.

### Establishing the Function of Spatio‐Temporal Enzyme(‐Like) Reactions in HMC

To increase the complexity in HMC for biomimetic reactions within and between different populations, cyclic enzyme‐induced pH switches, as in cells, are of great interest and a requirement for stimulating and shutting down membrane permeability for (intra‐)cellular reactions. The successful cyclic pH‐switches (pH 7.5 versus pH 6.4), induced by the sequential, time‐dependent addition of glucose and L‐phenylalanine to HMC (Figure S28b), also outline the high docking stability of Azo‐Psomes on the Coa@DMPC surface in HMC over the time again under continuously changing environmental conditions (Figure S28a). The results also indicate that the feedback‐controlled permeable and impermeable Azo‐Psomes membrane (Figure 2d) can be used for different scenarios of spatio‐temporal biomimetic reactions, which also means that the whole system of HMC is stable. The established principles of pH‐stable HMC for mimicking pH homeostasis, high docking stability of Azo‐Psomes within MHC, and feedback‐controlled pH‐switchability and loading capacity of Azo‐Psomes offer the opportunity to load Azo‐Psomes with other enzymes or enzyme‐like functions for switching on and off such (biomimetic) reactions in spatially separated nanocompartments, while cyclic pH switching takes place in the microcompartment of HMC (Scheme 1).

To demonstrate the occurrence of synergistic behaviors within pH‐responsive HMC under the consideration of cyclic pH switches (Scheme 1), (β‐cyclodextrin)_2_Hemin chloride complexes ((βCD)_2_Hemin) (Figure 6a) provide pH‐dependent peroxidase‐like behavior in the lumen of Psomes, proven by the oxidation of TMB (enzyme reaction substrate) into TMBox (enzyme reaction metabolite), known from a previous study.^[^ ^40^ ^]^ Hemin, a pH‐dependent biologically active iron porphyrin compound, in this structure features an analogue catalytic center of HRP, and β‐CD enhances the complex's solubility, making it easy to load this complex into Psomes. Azo‐Psomes (15 wt%), post‐loaded with (βCD)_2_Hemin ((βCD)_2_Hemin‐Azo‐Psomes) (Figures 6a, S29), outline expected pH‐dependent peroxidase‐like behavior (Figure 6d), considering artificial organelle. The pH‐dependent enzyme‐like behavior of (βCD)_2_Hemin‐Azo‐Psomes can be explained by the different state of membrane permeability. Briefly, at pH 7.4 membrane of (βCD)_2_Hemin‐Azo‐Psomes is in the collapsed state where TMB can only slowly penetrate the collapsed membrane (Figure 6a), and few (βCD)_2_Hemin complexes are captured in the membrane of Azo‐Psomes, while most complexes are integrated in the lumen of Azo‐Psomes. At pH 6 (βCD)_2_Hemin‐Azo‐Psomes are in the swollen state, stimulating a higher TMB membrane crossing for final conversion in the lumen. This diffusion behavior of TMB is supported by a recently published study,^[^ ^40^ ^]^ that (βCD)_2_Hemin complexes cannot be released from the lumen outside of Psomes. These diffusion characteristics of (βCD)_2_Hemin complexes can also be assumed in the present study, when finally considering the results of spatio‐temporal enzyme(‐like) reactions in HMC (Figure 6e).

The experimental setup for the spatio‐temporal enzyme(‐like) reactions, taking into account the previously obtained results confirming the required functional and structural principles of HMC, is presented in Figure 6a. It is assumed that the swelling and shrinking of (βCD)_2_Hemin‐Azo‐Psomes membranes are smoothly triggered by enzymatic induced cyclic pH switches in the GOx‐ and PAL‐loaded microcompartment of HMC by the sequential addition of glucose and L‐phenylalanine. The H_2_O_2_ produced during the conversion of glucose into gluconic acid by GOx enables (βCD)_2_Hemin complexes in Azo‐Psomes to provide the function of peroxidase‐like enzymes under pH‐dependent conditions. Assuming, (βCD)_2_Hemin complexes in Azo‐Psomes will not interfere with the desired Azo‐Psomes docking by inserting their azobenzene units in the phospholipid bilayer of the enzyme‐loaded microcompartment in HMC. Moreover, (βCD)_2_Hemin‐Azo‐Psomes outline similar pH‐responsiveness behavior (Figure 6b,c, eg., pH* of about 6.7) as presented for pure Azo‐Psomes (Figure 2d,e).

After 20 min incubation of (βCD)_2_Hemin‐Azo‐Psomes with Coa@DMPC microcompartment to fabricate pH‐responsive HMC, the environmental pH is adjusted to pH 7.4. The peroxidase‐like activity of (βCD)_2_Hemin‐Azo‐Psomes is very low in this environment after the addition of TMB (Figure 6d). The sequential ( = time‐dependent) addition of glucose (control experiment without the addition of L‐phenylalanine) causes the needed GOx‐converted glucose within Coa@DMPC to produce gluconic acid to decrease the environmental pH (Figure 6e). This instantly induces the swelling of (βCD)_2_Hemin‐Azo‐Psomes membrane to increase the peroxidase‐like activity of (βCD)_2_Hemin (Figure 6e) under the consumption of GOx‐produced H_2_O_2_ for the final TMB oxidation at acidic pH (Figure 6a). On the other hand, the sequential and cyclic addition of glucose and L‐phenylalanine to mimic pH homeostasis of HMC exhibits an increase of peroxidase‐like activity of (βCD)_2_Hemin‐Azo‐Psomes by glucose addition, while L‐phenylalanine almost shuts it down (Figure 6e). In this experiment series, the concentration of the substrates (glucose and L‐phenylalanine) is increased compared to the previous experiment in order to observe changes in the enzymatic (‐like) activity more rapidly.

The HMC for inducing spatio‐temporal enzymatic(‐like) activity (Scheme 1) outlines the expected synergistic effects, meaning the orchestration of functional and structural principles, between the different populations of nano‐ and microcompartment. The feedback‐controlled pH‐dependent swelling and deswelling of (βCD)_2_Hemin‐Azo‐Psomes in HMC are undoubtedly triggered by the spatio‐temporal control of diffusion processes by the proximity of docked nanocompartments on the microcompartment surface; achieving an accurate response under the transfer of metabolites from population to population, and from population to environment. This may help to understand synergistic behaviors between populations.

## Conclusion

In comparison to previous findings,^[^ ^11^, ^21^, ^24^, ^25^, ^26^ ^]^ the unification of distinct biomimetic functions in the formation of pH‐responsive hierarchically organized multi‐compartments (HMC) enables the occurrence of metabolite‐driven communication between two integrated populations within the HMC, ultimately leading to the initiation of (cyclic) spatio‐temporal peroxidase‐like reactions in a defined nanocompartment at the outer surface of the HMC.

The integration of biomimetic functions is crucial for stable pH‐ and nutrient‐responsive HMC. This is achieved by docking azobenzene‐containing polymersomes (Azo‐Psomes) to the surface of phospholipid bilayer‐membranized coacervates. Tight docking is based on azobenzene insertion into the cholesterol‐stabilized bilayer due to the presence of cholesterol moieties on the coacervate droplet surface.

The installation of biomimetic functions of pH homeostasis and artificial organelles (Azo‐Psomes) with storage and/or enzyme‐like functions in separate nano‐ and microcompartments in HMC is finally attributed to two different membrane diffusion barriers. The protonated membrane state of Azo‐Psomes is responsible for the delivery of metabolites or the execution of spatio‐temporal enzyme‐like reactions. Conversely, the deprotonated membrane state of Azo‐Psomes suppresses any delivery or enzyme‐like reactions. This enables pH‐dependent regulation in Azo‐Psomes through feedback mechanisms. In contrast, the phospholipid membrane of the microcompartment controls the size‐selective exchange of small molecules and nutrients between HMC populations and their environment. To conduct the biomimetic molecular communication of the nano‐ and microcompartment within HMC, it is necessary to integrate GOx, which is fed by the addition of glucose, and PAL, which is fed by the addition of L‐phenylalanine. This integration provides biomimetic pH homeostasis, finally leading to spatio‐temporal control of diffusion processes (Scheme 1). The proposed diffusion processes depend on direct docking of nanocompartments to the surface of microcompartments, and the kinetics of processes can be controlled by substrate loading. This phenomenon aligns with the proximity effect observed in numerous biological processes. In addition, similar pH regulation in unstable undocking systems requires more substrates and reaction time.

Cyclic, sequential, and out‐of‐equilibrium biological reactions are fundamental in living systems. These functions result from the integration of spatio‐temporal control of diffusion processes and enzyme‐like reactions within HMC, as shown in Scheme 1. To enable the final pH‐triggered peroxidase‐like reaction of (βCD)_2_Hemin‐Azo‐Psomes, it is crucial to maintain HMC stability during biomimetic pH homeostasis. The cyclic swelling and deswelling of Azo‐Psomes do not disrupt the microcompartment membrane, and no undocking of Azo‐Psomes occurs at acidic pH. Thus, HMC, capable of biomimetic pH homeostasis, remain stable across various conditions. This stability is crucial for triggering the peroxidase‐like activity of (βCD)_2_Hemin‐Azo‐Psomes.

Overall, the established HMC clearly demonstrates that cooperative behavior between different populations can be used to achieve biomimetic biological tasks for higher complexity. It presents a novel approach for non‐covalent interactions and synergistic effects among artificial nano‐ and microcompartments, thereby facilitating feedback‐controlled behaviors under the influence of spatio‐temporally controlled diffusion processes and enzyme(‐like) reactions. Moreover, the surface docking potential of HMC makes the diffusion and reaction behaviors more efficient and controllable. This demonstrates new concepts for enhancing the complexity of artificial cell compartments in their collective behaviors (e.g., the assembly of differently composed HMC by host‐guest interactions). This opens a promising avenue for elucidating the mechanisms underlying synergistic and responsive behaviors with real cells.

## Conflict of Interests

The authors declare no conflict of interest.

## Supporting information



Supporting Information

## Data Availability

The data that support the findings of this study are available in the supplementary material of this article.
