# Supplementary material for: Spatio‐Temporal Processes of Diffusion‐Controlled Communication in Hierarchical Multi‐Compartments
Source: Angew Chem Int Ed Engl. 2025 May 8;64(26):e202424133. doi: 10.1002/anie.202424133 (PMC12184289; doi:10.1002/anie.202424133)
Supplement: Supplementary file 1 — Supporting Information [file ANIE-64-e202424133-s001.docx]

**SUPPORTING INFORMATION**

**Spatio-temporal processes of diffusion-controlled communication in hierarchical multicompartments**

Xin Qiao,^a^ Haixu Chen,^a^ Andreas Schurig,^b^ Xiaoliang Wang,^a^ Yinyong Sun,^a^ Matthias Tobler,^c^ Susanne Boye,^b^ Kathrin Castiglione,^c^ Dietmar Appelhans*^b^ and Xin Huang*^a^

^a^ MIIT Key Laboratory of Critical Materials Technology for New Energy Conversion and Storage, School of Chemistry and Chemical Engineering, Harbin Institute of Technology, Harbin 150001, China

^b^ Leibniz-Institut für Polymerforschung Dresden e.V., Hohe Straße 6, Dresden 01069, Germany

^c^ Institute of Bioprocess Engineering, Friedrich-Alexander-Universität Erlangen-Nürnberg, Paul-Gordan-Straße 3, 91052 Erlangen

*Corresponding authors: [applhans@ipfdd.de](mailto:applhans@ipfdd.de); [xinhuang@hit.edu.cn](mailto:xinhuang@hit.edu.cn)

**Table of content**

[1. Material 4](#_Toc191919232)

[2. Methods 5](#_Toc191919233)

[2.1 Nuclear magnetic resonance (NMR) spectroscopy 5](#_Toc191919234)

[2.2 Size exclusion chromatography (SEC) 5](#_Toc191919235)

[2.3 Hollow fiber filtration (HFF) 5](#_Toc191919236)

[2.4 Dynamic light scattering (DLS) 6](#_Toc191919237)

[2.5 Zeta potential (ζ) 6](#_Toc191919238)

[2.6 UV lamp for crosslinking of Azo-Psomes 6](#_Toc191919239)

[2.7 UV–Vis spectroscopy 7](#_Toc191919240)

[2.8 Confocal laser scanning microscopy (CLSM) 7](#_Toc191919241)

[2.9 Asymmetrical flow field-flow fractionation (AF4) 7](#_Toc191919242)

[3. Experimental part 9](#_Toc191919243)

[3.1 Synthesis of succinylated amylose^[2]^ 9](#_Toc191919244)

[3.2 Synthesis of methacryloxyethyl dimethylethyl ammonium bromide (MEDAB)^[3]^ 9](#_Toc191919245)

[3.3 Synthesis of cholesteryl acryloyoxy ethyl carbonate^[4]^ 10](#_Toc191919246)

[3.4 Synthesis of poly (methacryloxyethyl dimethylethane ammonium bromide-co-cholesterol-modified acrylate (PMEDAB-Chol)^[5]^ 10](#_Toc191919247)

[3.5 Synthesis of the general block copolymers, BCP A and RhB-BCP A, by ATRP 11](#_Toc191919248)

[3.6 Synthesis of Azo-BCP by one pot of ATRP and Click reaction 11](#_Toc191919249)

[3.7 Synthesis of dye-labeled BCP A 12](#_Toc191919250)

[3.8 Preparation of (βCD)_2_Hemin Stock solution (2 mg/mL) 13](#_Toc191919251)

[3.9 Preparation and crosslinking of Azo-Psomes 13](#_Toc191919252)

[3.10 Fabrication of post loaded (βCD)_2_Hemin-Azo-Psomes 14](#_Toc191919253)

[3.11 Preparation of Coa@DMPC 15](#_Toc191919254)

[3.12 Reversible swelling of Azo-Psomes upon repeating changes in pH in 1 mM PBS 15](#_Toc191919255)

[3.13 Study on the docking of Azo-Psomes with Coa@DMPC 16](#_Toc191919256)

[3.14 Labeling GOx and PAL with fluorescence dyes (Cy5-GOx and Cy7-PAL) 16](#_Toc191919257)

[3.15 Expression and purification of the phenylalanine ammonia lyase (PAL) 16](#_Toc191919258)

[3.16 Deamination of L-Phenylalanine with PAL to test enzyme activity at different pH 18](#_Toc191919259)

[3.17 FACS analysis 18](#_Toc191919260)

[3.18 Study on GOx enzyme activity loaded by Coa@DMPC 19](#_Toc191919261)

[3.19 Study on PAL enzyme activity loaded by Coa@DMPC 19](#_Toc191919262)

[3.20 Study on the fluorescence changes of FITC in Coa@DMPC loaded with GOx after adding glucose 20](#_Toc191919263)

[3.21 Study on the docking of Azo-Psomes with Coa@DMPC by CLSM 20](#_Toc191919264)

[3.22 Study on the one-time release of L-phenylalanine in Azo-Psomes 21](#_Toc191919265)

[3.23 Study on the glucose-triggered enzymatic reaction between Coa@DMPC and Azo-Psomes 21](#_Toc191919266)

[3.24 Study on the glucose-triggered enzymatic reaction between coacervate droplets and Azo-Psomes 22](#_Toc191919267)

[3.25 Study on the docking of Azo-Psomes on Coa@DMPC surface under pH cycle changes 22](#_Toc191919268)

[3.26 Study on the docking of Azo-Psomes on Coa@DMPC surface after pH reduction 23](#_Toc191919269)

[3.27 TMB assay at different pH values of (βCD)_2_Hemin-Azo-Psomes 23](#_Toc191919270)

[3.28 Regulation of peroxidase-mimicking activity of (βCD)_2_Hemin-Azo-Psomes 24](#_Toc191919271)

[4. Additional Schemes, Figures and Tables 25](#_Toc191919272)

[5. References 47](#_Toc191919273)

# Material

Amylose (M_w_ = 12000 g/mol, Carbonsynth), FITC (Fluorescein isothiocyanate isomer I, Sigma 90%), RBITC (Rhodamine B isothiocyanate, Sigma), 2-(N,N-dimethylamino)ethyl methacrylate (DMAEMA, Energy Chemical), (α-Azide, ω-hydroxy)-terminated poly (ethylene glycol) (N_3_-PEG_63_-OH, Mn = 2.8 kDa, Polymer Source), 2-(N,N-diethylamino)ethyl methacrylate (DEAEMA, TCI), copper(I) bromide (CuBr, Sigma), bromoethane (BE, Energy Chemical, 99%), cholesteryl chloroformate (Energy Chemical), triethylamine (Energy Chemical), 2-hydroxyethyl acrylate(Energy Chemical), 2,2'-azobis(2-methylpropionitrile) (AIBN, Energy Chemical), 1-acyl-2-{6-[(7-nitro-2-1,3-benzoxadiazol-4-yl)amino]hexanoyl}-sn-glycero-3-phosphocholine (NBD-PC, Avanti), 1,2-dimyristoyl-sn-glycero-3-phosphocholine (DMPC, Aladdin), 4-(dimethylamino) pyridine (DMAP, Sigma), glucose oxidase (GOx, Sigma), D-(+)-glucose (≥ 99.5%, Sigma), L-Phenylalanine (Sigma), succinic anhydride (Sigma), hemin chloride (3ABio, 97%), 3,3',5,5'-tetramethylbenzidine (TMB, Energy Chemical, 98%), beta-cyclodextrin (β-CD, Energy Chemical, 99%), , peptone from casein (Merck), yeast extract (Carl Roth), NaCl (Carl Roth, ≥99.5%), agar-agar (Carl Roth, bacteriological), Na_2_HPO_4_ (Carl Roth, ≥98%), KH_2_PO_4_ (Carl Roth, ≥99%), NH_4_Cl (Carl Roth, ≥99.7%), Na_2_SO_4_ (Carl Roth, ≥99%), MgSO_4_ (Carl Roth, ≥99%), glycerol (Carl Roth, ≥99.5%), glucose (Carl Roth, for microbiology), lactose (Carl Roth, for microbiology), FeCl_3_ ∙ 6H_2_O (Thermo Scientific, ≥98%), CaCl_2_ ∙ 2H_2_O (Carl Roth, ≥99%), MnSO_4_ ∙ H_2_O (Carl Roth, ≥99%), ZnSO_4_ ∙ 7H_2_O (Sigma, ≥99.95%), CoCl_2_ ∙ 6H_2_O (Carl Roth, ≥99%), CuCl_2_ ∙ 2H_2_O (Sigma, ≥99.95%), NiCl_2_ ∙ 6H_2_O (Carl Roth≥98%), Na_2_MoO_4_ ∙ 2H_2_O (Sigma, ≥99.5%), Na_2_SeO_3_ (Carl Roth, ≥99%), H_3_BO_3_ (Merck, ≥99.5%), carbenicillin (Carl Roth, ≥88%), TRIS-HCl (Carl Roth, ≥99%), and imidazole (Carl Roth, ≥99%) were purchased from different companies. Milli-Q water was employed to prepare all the solutions and PBS buffer was prepared by PBS tablet (Carl Roth, Karlsruhe, Germany). All dialysis membranes, nylon filter (0.1, 0.2 µm) and cellulose mixed ester filter (CME, 0.8 µm) were purchased from Carl Roth (Karlsruhe, Germany).

# 2. Methods

## 2.1 Nuclear magnetic resonance (NMR) spectroscopy

The chemical shifts of block copolymers and small chemical molecules were measured on 500 MHz Bruker Avance III 500 spectrometer (^1^H NMR, Bruker Biospin MRI GmbH, Ettlingen, Germany) in D_2_O (D_2_O, δ = 4.80 ppm).

## 2.2 Size exclusion chromatography (SEC)

The weight average molecular weight (*M*_w_), the number of average molecular weight (*M*_n_), and molar mass distributions (*Ð*, *M*_w_/*M*_n_) of all copolymers were determined by SEC equipped with a MALLS detector (Mini DAWN-LS detector, Wyatt Technology Corp., Santa Barbara, CA, USA) and a viscosity/refractive index (RI) detector (ETA-2020, WGE Dr Bures GmbH&Co KG, Dallgow-Doeberitz, Germany). The column (PL MIXED-C with a pore size of 5 μm, 300x7.5 mm) and the pump (HPLC pump, Agilent 1200 series) were from Agilent Technologies (Santa Clara, CA, USA). DMAc was used as an eluent (mixed with 2 v% water, 3 g/L LiCl) with a flow rate of 0.5 mL/min for all block copolymers. The calibration was based on poly(2-vinylpyridine) standards. The data were processed using Cirrus GPC offline GPC/SEC software version 2.0 (Agilent Technologies, Santa Clara, CA, USA).

## 2.3 Hollow fiber filtration (HFF)

Purification of pure Azo-Psomes solutions and Azo-Psomes, loaded with L-Phenylalanine, were carried out via HFF using KrosFlo Research Iii System (BioProcess International, New York, NY, USA). It was equipped with a separation module (polyether sulfone membrane) with MWCO of 750 kDa, (Repligen.com, California, USA). The transmembrane pressure was set at 0.10 bar, and flow rate was 15 mL/min. All Psomes solutions were purified by washing continuously with Milli-Q water.

## 2.4 Dynamic light scattering (DLS)

DLS tests of RhB-Azo-Psomes, Azo-Psomes or (βCD)_2_Hemin-Azo-Psomes solutions were measured by a Zetasizer Nano-series instrument equipped with Dispersion Technology Software version 5.00 (Malvern Instruments, Malvern, UK). The measurements were carried out at 25°C. The data was collected using the NIBS (non-invasive back-scatter) method using a Helium-Neon laser (4 mW, λ = 633 nm) and a fixed angle of 173°. Viscosity is 0.8872 cP and RI is 1.33. The equilibration is 120 s, and disposable cuvettes were used. Measurement duration was automatic, number of runs were 11, and run duration was 10 s. Number of measurements was three times. The data were analyzed using Malvern Software 7.13 (Malvern Instruments, Malvern, UK), using cumulant´s fit for the autocorrelation curves.

## 2.5 Zeta potential (ζ)

Zeta potenrial of Su-Am, P-Chol or Coa@DMPC solutions were measured by a Zetasizer Nano-series instrument equipped with Dispersion Technology Software version 5.00 (Malvern Instruments, Malvern, UK). The measurements were carried out at 25°C. The data was collected using the NIBS (non-invasive back-scatter) method using a Helium-Neon laser (4 mW, λ = 633 nm) and a fixed angle of 173°. Viscosity is 0.8872 cP and RI is 1.33. The equilibration is 120 s, and potential sample pool were used. Measurement duration was automatic, number of runs were 11, and run duration was 10 s. Number of measurements was three times. The data were analyzed using Malvern Software 7.13 (Malvern Instruments, Malvern, UK).

## 2.6 UV lamp for crosslinking of Azo-Psomes

Crosslinking of Azo-Psomes was carried out using a high-pressure mercury lamp as a UV source in EXFO Omnicure 2000 (Lumen Dynamics GroupInc., Mississauga, Ontario, Canada). The crosslinking process was performed with 1.5 mL of Azo-Psomes solution for 50 s.

## 2.7 UV–Vis spectroscopy

UV–Vis analysis was performed using Specord 210 Plus double beam UV-Vis spectrophotometer (analytikjena, Jena, Germany). Samples were measured at desired wavelength range in semi-micro cuvettes (Brand GmbH + CO KG, Wertheim, Germany).

## 2.8 Confocal laser scanning microscopy (CLSM)

CLSM images were obtained on a Leica SP 8 equipped with an UV-Diode (405 nm), an Argon (488 nm), a DPSS (552 nm), and a HeNe (638 nm) laser, using a 40x oil immersion objective, and acquisition processing by Leica LAS AF software (Leica Microsystems, Wetzlar, Germany). The used objectives were 40x/1.25-0.7 oil. The excitation was caused by the Argon laser and filters (488 nm), DPSS (552 nm), HeNe laser and filters (638 nm). Prepared samples, described below, were used by dropping 5-10 µL of fluorescently labelled solution onto a glass slide.

## 2.9 Asymmetrical flow field-flow fractionation (AF4)

Based on previous studies^[1]^ AF4-LS measurements were conducted on Eclipse Neon (Wyatt Technologies Europe) equipped with a Dilution Control Module^TM^ (DCM) using an Agilent pump system (1260, Infinity Series) at 25 °C in PBS buffer (1 mM, pH 7.4) containing 200 mg/L NaN_3_ as carrier liquid to prevent bacteria or algae contamination. A short channel with fixed height (350 μm) and regenerated cellulose

(cut-off: 10 kDa) as ultrafiltration membrane for all measurements. Detection was performed using LS detector (DAWN Neon, Wyatt λ = 660 nm) with QELS option, RI detector (Optilab T-rEX, Wyatt) and UV detectors (λ = 280 nm). The following protocol was applied (**Figure S1**): channel flow was set to 1.0 mL/min with dilution control module split of 1:1 resulting in a detector of 0.5 mL/min, focusing was performed with focus flow (F_f_) 2.0 mL/min for 5 min followed by an isocratic elution step with an F_x_ of 2 mL/min for 3 min followed by an exponential F_x_ gradient from 2 to 0.05 mL/min within 30 min and a linear gradient from 0.05 to 0 mL/ min within 10. The last step proceeds without F_x_ (0 mL/min) for 10 min. Injections of three times 30 μL of each sample were performed using an autosampler (1260 series, Agilent Technologies Deutschland GmbH, Waldbronn, Germany). Data was collected and processed using Astra Version 8.1.2. M_w_ and radius of gyration (R_g_) of Psomes were calculated from the MALS data of detectors 6 to 17 applying Berry fit. Dn/dc was determined previously (dn/dc = 0.185 mg/mL).

**Figure S1.** Optimized flow profile for AF4 separation.

**2.10 Cryogenic transmission electronic microscopy (cryo-TEM)**

Cryo-TEM images were acquired using the FEI Talos F200C microscope (Thermo Fisher Scientific, America) at an acceleration voltage of 220 kV. The hydrophilized copper mesh was placed into the FEI Vitrobot device with tweezers, a certain amount of Azo-Psomes (generally 3-5 microliters) was dropped on the copper mesh, and after Waiting time, most of the sample solution was absorbed with filter paper (Blot time), and the copper mesh was quickly put into liquid ethane for quick freezing. The quick-frozen copper mesh is stored in a TEM four-hole storage box cooled by liquid nitrogen for testing. The diameter and membrane thickness of the Azo-Psomes were determined from cryo-TEM images by using ImageJ Software

# 3. Experimental part

## 3.1 Synthesis of succinylated amylose^[2]^

Succinylated amylose (Su-Am) was prepared by dissolving 100 mg of amylose (8.3 μmol) and 185 mg of succinic anhydride (1.85 mmol) in 15 mL of DMSO at 60°C. After complete dissolution of the amylose, 5 mg of DMAP (0.04 mmol) was added and the reaction mixture was left to stir for 16 h. After the reaction, the mixture was diluted with 30 mL water and dialyzed extensively against water using a dialysis tube with MWCO of 3.5 kDa. (75.3 mg, yield: 75%) (**Figure S2**).

**^1^H NMR** (500 MHz, D_2_O): δ_H_ (ppm) = 2.4-3.0 and 5.3-5.8 (m, glucose protons per units), 3.79-3.47 (m, HOOC-CH_2_-CH_2_-COOR, succinyl).

## 3.2 Synthesis of methacryloxyethyl dimethylethyl ammonium bromide (MEDAB)^[3]^

2-(N,N-dimethylamino) ethyl methacrylate (DMAEMA, 10 g, 0.06 mol) and bromoethane (4.6 g 0.04 mol) with mole ratio of 1:1.3 were dissolved in 30 mL of acetone in a round bottom flask. The reaction was carried out at 50°C for 5 h under the protection of N_2_. After stopping the reaction, excess solvents were removed by vacuum distillation at 50°C. Then the yellowish viscous coarse product was purified by precipitation in anhydrous diethylether for three times. For the precipitation the crude product was dissolved in 200 mL of diethyl ether. After drying in vacuum at room temperature, final product was gained as white powder (8.2 g, yield: 54.6%) (**Figure S3**).

**^1^H NMR** (500 MHz, D_2_O): δ_H_ (ppm) = 1.42 (t, N^+^-CH_2_-CH_3_), 1.91 (s, -CO-C (CH_3_)=CH_2_), 3.46 (m, -(CH_2_)-N^+^-(CH_3_)_2_), 4.12 (q, N^+^-CH_2_-CH_3_), 4.12 (m, -COO-CH_2_-CH_2_-N^+^), 4.62 (m, -COO-CH_2_-CH_2_-N^+^), 5.64 and 6.10 (s, -CO-C(CH_3_)=CH_2_).

## 3.3 Synthesis of cholesteryl acryloyoxy ethyl carbonate^[4]^

1.125 g of cholesteryl chloroformate (2.5 mmol) was added to a 100 mL triple-bottomed round-bottomed flask and the sample was dissolved in 10 mL of dichloromethane, followed by the addition of 0.2777 g of triethylamine (2.74 mmol) and an ice bath. Subsequently, 0.4375 g of 2-hydroxyethyl acrylate (6.07 mmol) diluted with 5 mL of dichloromethane was slowly added dropwise to the mixture and the system was allowed to react for 6 h at room temperature. At the end of the reaction, the reaction solution was progressively extracted with 20 mL of HCl (0.5 M), 20mL of NaHCO_3_ solution (pH 8.5), 20mL of H_2_O, and 20mL of saturated NaCl solution. After adding anhydrous Na_2_SO4 and filtering, the product was obtained after spinning and vacuum drying (0.82 g, yield: 62.1%) (**Figure S4**).

**^1^H NMR** (500 MHz, D_2_O): δ_H_ (ppm) =0.5-2.5 (m, cholesterol), 4.46 (d, -O-CH_2_-CH_2_-O-), 4.50 (m, H of hexatomic ring), 5.46 (m, -C-CH- of hexatomic ring), 5.93 (d, CH_2_-CH-), 6.21 (m, CH_2_-CH-),6.49 (m, CH_2_-CH-).

## 3.4 Synthesis of poly (methacryloxyethyl dimethylethane ammonium bromide-co-cholesterol-modified acrylate (PMEDAB-Chol)^[5]^

Mercaptothiazoline-activated trithiol-RAFT agent (6.8 mg, 14.7 μmol), AIBN (1 mg, 6.0 μmol), cholesterol formylacrylate ethyl ester (80 mg, 0.15 mmol), MEDAB (220 mg, 1.15 mmol) and DMF (4 mL) were added to a 10 mL of round-bottom flask. The polymerization was carried out at 65°C for 9 h, and purified by two times precipitation in diethyl ether/hexane (1:2 volume ratio), finally, it is precipitated once in trichloromethane (163.4 mg, yield: 53%) (**Figure S5**).

**^1^H NMR** (500 MHz, D_2_O): δ_H_ (ppm) = 0.7-2.0 (m, cholesterol), 3.13 (m, -(CH_2_)-N^+^-(CH_3_)_2_), 3.75 (m, -COO-CH_2_-CH_2_-N^+^), 4.43 (d, -COO-(CH_2_)_2_-O-), 4.51 (d, -COO-CH_2_-CH_2_-N^+^), 5.72 (d, -CH=C- of six-membered ring).

## 3.5 Synthesis of the general block copolymers, BCP A and RhB-BCP A, by ATRP

BCP A and RhB-BCP A were synthesized followed by previously published procedure (**Scheme S1**).^[6]^ BCP A is the standard BCP for the fabrication of various pH- and redox-responsive polymersomes.^[6]^ Molecular compositions, determined by ^1^H NMR (**Figures S7, S9**), and molecular weight, determined by SEC, of BCP A are presented in **Table S1**.

BCP A:**^1^H NMR** (500 MHz, CDCl_3_): δ_H_ (ppm) = 2.57 (4, R-N(-CH_2_-CH_3_)_2_, 2.7 (3, R_2_N-CH_2_-CH_2_-OR), 3.38 (6, CH_3_-PEG), 3.52 (2, N-CH_2_ from photo-crosslinker), 3.65 (1, PEG), 3.93 + 4.0 (5, COO-CH_2_).

RhB-BCP A:**^1^H NMR** (500 MHz, CDCl_3_): δ_H_ (ppm) = 2.57 (4, RN(-CH_2_-CH_3_)_2_, 2.7 (3, R_2_N-CH_2_-CH_2_-OR), 3.38 (6, CH_3_-PEG), 3.52 (2, N-CH_2_ from photo-crosslinker), 3.65 (1, PEG), 3.93 + 4.0 (5, COO-CH_2_), 6.25-6.75 (RhB, 9 aromatic H).

## 3.6 Synthesis of Azo-BCP by one pot of ATRP and Click reaction

The synthesis of azo-benzene-containing amphiphilic block copolymer (Azo-BCP, azobenzene-polyethyleneglycol-*b*-poly(diethylaminoethylmethacrylate-co-(3,4-dimethylmaleimido) butyl methacrylate)) and requested educts was realized by previously published procedures (**Scheme S1**).^[7]^ The requested educts to be synthesized for the atom transfer radical polymerization (ATRP) and click reaction were the photo-crosslinker dimethylmaleinimidobutyl methacrylate (DMIBMA), 2-(N,N-diethylamino)ethyl methacrylate (DEAEMA), azido-functionalized macroinitiator for ATRP, and propargyl-modified azobenzene (Azo-PP) for click reaction.^[7]^

Azo-BCP was synthesized by a one-pot reaction for ATRP and click reaction. In a dried 25 mL Schlenk flask, AZO-PP (14.16 mg, 0.06 mmol), macroinitiator N_3_-PEG_63_-Br (140 mg, 0.05 mmol), 2,2’-bipyridine (14.5 mg, 0.093 mmol), monomer DEAEMA (0.655 mL, 3.26 mmol), DMIBMA (247 mg, 0.93 mmol) and dry 2-butanone (1.5 mL) were added under a N_2_ atmosphere. After the reaction flask was degassed via two freeze-pump-thaw cycles, CuBr (6.7 mg, 0.047 mmol) was added. The system was degassed via one freeze-pump-thaw cycle again and, then, placed in an oil bath preheated at 50°C for 18 h. The reaction mixture was diluted with THF and passed through a neutral alumina column to remove the catalysts. The mixture was concentrated and dialyzed for two days against acetone using dialysis membrane (MWCO 5 kDa) to remove unreacted AZO-PP, macroinitiator and monomer. After removing the solvent by rotary evaporation and vacuum drying, a dark yellow sticky block copolymer was collected and isolated of about 740 mg. Molecular composition, determined by ^1^H NMR (**Figures S8**), and molecular weight, determined by GPC, of Azo-BCP are presented in **Table S1**.

**^1^H NMR** (500 MHz, CDCl_3_): δ_H_ (ppm) = 2.57 (4, RN-CH_2_-CH_3_)_2_, 2.7 (3, R_2_N-CH_2_-CH_2_-OR), 3.52 (2, N-CH_2_ from photo-crosslinker), 3.65 (1, PEG), 3.93 + 4.0 (5, COO-CH_2_), 4.57 (6, t, triazole-CH_2_), 5.31 (8, s, O-CH_2_-triazole), 7-8 (9, aromatic H), 7.88 (7, s, -CH- of triazole).

## 3.7 Synthesis of dye-labeled BCP A

For CLSM study, two different dye-labeled BCP A were realized: (i) rhodamine B-labeled BCP A (RhB-BCP A) and (ii) cyanine 5-labeled BCP A (Cy5-BCP A). RhB-BCP A was established by a previously published description.^[7]^ Cy5-BCP A was established by the synthesis of pyridyldisulfidethyl-modified BCP (PDSMA-BCP A) through RAFT polymerization, followed by the final thiol-maleimide reaction through the conversion of PDSMA-BCP A with Cy5-maleimido in presence of TCEP (tris-carboxyethylphosphine) at which pyridylthiol is released from PDSMA-BCP A to undergo desired thiol-maleimide reaction in MeOH at 30°C. Chemical structures of both BCPs, RhB-BCP A and Cy5-BCP A, are presented in **Schemes S1** and **S2**, respectively. Molecular composition, determined by ^1^H NMR (**Figures S9, S22**), and molecular weight, determined by SEC, of RhB-BCP A and PDSMA-BCP A are presented in **Table S1**.

PDSMA-BCP A:**^1^H NMR** (500 MHz, CDCl_3_): δ_H_ (ppm) = 2.57 (4, RN(-CH_2_-CH_3_)_2_, 2.7 (3, R_2_N-CH_2_-CH_2_-OR), 3.05 (8, R-S-S-CH_2_-CH_2_-OR), 3.38 (6, CH_3_-PEG), 3.52 (2, N-CH_2_ from photo-crosslinker), 3.65 (1, PEG), 3.93 + 4.0 (5, COO-CH_2_), 4.25 (7, COO-CH_2_-CH_2_-S_2_-R), 7.13 (9, aromatic H), 7.69 (10+11, aromatic H), 8.48 (12, aromatic H).

## 3.8 Preparation of (βCD)_2_Hemin Stock solution (2 mg/mL)

A solution of (βCD)_2_Hemin was prepared by dissolving Hemin chloride (4 mg, 6.13 μmol) and 13.9 mg (0.12 mmol) of β-CD in 2 mL NaOH (0.1 M) and stirring 1 h at 37℃. The resulting solution was completely soluble. The solution was filtrated using 0.2 μm nylon filter and stored in the fridge at 4°C for further use.

## 3.9 Preparation and crosslinking of Azo-Psomes

***Azo-Psomes (15wt%):*** The BCP A and Azo-BCP were respectively dissolved in diluted 0.01 M HCl at pH 2 and the concentration was 1.0 mg/mL for each BCP. The final mixture solution of 8.5 mL of BCP A and 1.5 mL of Azo-BCP was passed through a syringe filter (Nylon, 0.2 μm), and then the pH was adjusted to 8.0-8.3 with 0.1 M NaOH. The sample was stirred in the dark for three days, passed through a 0.8 μm syringe filter, and crosslinked in small aliquots (1.5 mL) for 50 s each by UV chamber.

***Azo-Psomes (25 wt%):*** The final mixture solution contained 7.5 mL of BCP A and 2.5 mL of Azo-BCP. Other conditions remained unchanged, including crosslinking time.

***RhB-Azo-Psomes*** or ***Cy5-Azo-Psomes*** (15 wt% of Azo-BCP, labeled by RhB-BCP A or Cy5-BCP A): The BCP A, RhB-BCP A, and Azo-BCP were respectively dissolved in diluted 0.01 M HCl at pH 2 and the concentration of each component was 1.0 mg/mL. The final mixture solution, consisting of 7.5 mL of BCP A, 1 mL of RhB-BCP A or Cy5-BCP A and 1.5 mL of Azo-BCP, was passed through a syringe filter (Nylon, 0.2 μm), and then the pH was adjusted to 8.0-8.3 with 0.1 M NaOH. The sample was stirred in the dark for three days, passed through a 0.8 μm syringe filter, and crosslinked in small aliquots (1.5 mL) for 50 s each by UV chamber.

***RhB-Azo-Psomes*** or ***Cy5-Azo-Psomes*** (25 wt% of Azo-BCP, labeled by RhB-BCP A or Cy5-BCP A): the mixture solution was 6.5 mL of BCP A, 1 mL of RhB-BCP A or Cy5-BCP A, and 2.5 mL of Azo-BCP. Other conditions remained unchanged, including crosslinking time.

***Azo-Psomes (15 wt%) loaded with L-Phenylalanine:*** The BCP A and Azo-BCP were dissolved respectively in diluted 0.01 M HCl at pH 2, and the concentration was 1.0 mg/mL for each BCP. The final mixture solution of 8.5 mL of BCP A and 1.5 mL of Azo-BCP was passed through a syringe filter (Nylon, 0.2 μm), and then the pH was adjusted to 6.0 with 0.1 M NaOH. The filtered 2 mL of L-phenylalanine solution (1.5 mg/mL) was added dropwise into the mixed solution and stirred for 30 min, and pH was adjusted to 8.0. The sample was stirred in the dark for three days, passed through a 0.8 μm syringe filter, and crosslinked in small aliquots (1.5 mL) for 50 s each by UV chamber. HFF was used to remove the free L-Phenylalanine not loaded in the Azo-Psomes. The loading efficiency of L-Phenylalanine after optimization with Azo-Psomes was 82%.

## 3.10 Fabrication of post loaded (βCD)_2_Hemin-Azo-Psomes

For post loading process, 2.925 mL of 1 mM PBS at pH 6, 75 μL of (βCD)_2_Hemin stock solution (2 mg/mL) was mixed with 3 mL of pure Azo-Psomes (15 wt%) solution. The sample was stirred for 24 h. After 24 h of post loading, the pH was adjusted to pH 5 and centrifuged twice in 6000 (RPM) for 4 min. After centrifugation, the pH of the supernatant was adjusted to 8 for the procedure of dialysis. Dialysis of supernatant was carried out against 1 mM PBS at pH 7.4 for 96 h in the dark to remove all free (βCD)_2_Hemin from unpurified loaded Azo-Psomes. Finally, the final concentration of (βCD)_2_Hemin-Azo-Psomes was made to be slightly 1 mg/mL by ultrafiltration.

## 3.11 Preparation of Coa@DMPC

20 mg/mL of Su-Am stock solution was diluted to 1mg/mL and 5 mg/mL of PMEDAB-Chol stock solution was diluted to 2 mg/mL. The coacervate droplets were prepared by mixing the polyelectrolyte solutions by volume ratio of 1:1. The final solution volume was 60 μL. After 20 min, 5 μL of phospholipid solution (20 mg/mL) was added. This final Coa@DMPC solution (65 µL) was then used for all further experiments related to the docking experiments with Azo-Psomes and pH-stability of Azo-Psomes on Coa@DMPC surface.

For FACS analysis final 140 µL of coarcervate droplets consisted of 1:1 volume ratio between both polyelectrolytes, while Coa@DMPC with FITC staining consisted of 120 µL of coacervate (1:1 volume), 10 µL of stained DMPC solution (5% NBD-PC) and 12 µL of FITC (stock solution, 1 mg/mL, H_2_O).

***FITC-Coa@DMPC*** or ***RhB-Coa@DMPC:*** Added 6 µL of FITC stock solution (1 mg/mL, H_2_O) or 6 µL of RhB stock solution (1 mg/mL, H_2_O) in 65 µL of Coa@DMPC to obtain the labelled Coa@DMPC solutions.

## 3.12 Reversible swelling of Azo-Psomes upon repeating changes in pH in 1 mM PBS

An Azo-Psomes (15 wt%), Azo-Psomes (25 wt%) or (βCD)_2_Hemin-Azo-Psomes (15 wt%) solution (0.25 mg/mL) in 1 mM PBS was prepared, and little amounts of 0.1 M HCl or 0.1 M NaOH were added to reach pH 4 or 8, respectively. The swelling and deswelling processes of the various Azo-Psomes solutions were investigated by DLS. pH-responsiveness of Azo-Psomes were followed by pH titration experiment from basic to acidic pH to finally determine the starting point of swelling, pH* (half power of swelling), and completely swollen state of Azo-Psomes by DLS.

## 3.13 Study on the docking of Azo-Psomes with Coa@DMPC

10 μL of FITC-Coa@DMPC solution was incubated with RhB-Azo-Psomes (15 wt% or 25 wt%) (1 mg/mL, 5 μL) for 15 min and the docking status was observed by CLSM. This volume ratio was 2:1. Other volume ratios were tested according to this method (1:1 = 10 μL of FITC-labelled Coa@DMPC:10 μL of RhB-Azo-Psomes; 10:1 = 10 μL of FITC-Coa@DMPC:1 μL of RhB-Azo-Psomes).

## 3.14 Labeling GOx and PAL with fluorescence dyes (Cy5-GOx and Cy7-PAL)

In a flask, GOx (20.0 mg, 0.125 μmol) was dissolved into PBS buffer solution (4.0 mL, 0.1 mM, pH 7.4). Then, 20 µL of Cy5-NHS DMSO solution (10.0 mg/mL, 1.28 μmol) was added dropwise. The solution was stirred at room temperature for 12 h, purified by dialysis membrane (MWCO 10 kDa), dialyzing against Milli-Q water for two days, and freeze-dried.

In an Eppendorf tube, PAL (4.2 mg, 0.084 μmol) was dissolved into TRIS-HCl buffer solution (750 µL, 2 mM, pH 8.8). Then 5 µL of Cy7-NHS in DMSO solution (10.0 mg/mL, 1.22 μmol) was added dropwise. The solution was stirred at room temperature for 12 h, purified by dialysis membrane (MWCO 50 kDa), dialyzing against Milli-Q water for two days, and freeze-dried.

## 3.15 Expression and purification of the phenylalanine ammonia lyase (PAL)

The plasmid pET22b-*At*PAL2 encoding the A*rabidopsis thaliana* phenylalanine ammonia lyase (PAL)^[8]^ was transformed into chemically competent *Escherichia coli* NiCo21 (DE3) via heat shock. Afterwards, the cells were regenerated in lysogeny broth (LB) medium (10 g/L peptone, 5 g/L yeast extract, 10 g/L NaCl) and plated out on LB agar (10 g/L peptone, 5 g/L yeast extract, 10 g/L NaCl, 15 g/L agar-agar) containing 100 mg/L carbenicillin.

For protein expression, cultivations were carried out in ZYM-5052 autoinduction medium adapted from Studier^[9]^ containing 1% peptone from casein, 0.5% yeast extract, 25 mM Na_2_HPO_4_, 25 mM KH_2_PO_4_, 50 mM NH_4_Cl, 5 mM Na_2_SO_4_, 2 mM MgSO_4_, 0.5% glycerol, 0.05% glucose, 0.2% lactose, 10 µM FeCl_3_, 4 µM CaCl_2_, 2 µM MnSO_4_, 2 µM ZnSO_4_, 0.4 µM CoCl_2_, 0.4 µM CuCl_2_, 0.4 µM NiCl_2_, 0.4 µM Na_2_MoO_4_, 0.4 µM Na_2_SeO_3_, and 0.4 µM H_3_BO_3_, as done previously (DOI: 10.1039/D3RE00056G). First, a 100 mL flask with 20 mL LB medium containing 100 mg/L carbenicillin was inoculated from a single colony and incubated for 8 h at 37 °C and 180 rpm. Then, a 1 L shake flask was inoculated from that containing 180 mL autoinduction medium with 100 mg/L carbenicillin. The cultures were first cultivated at 37 °C for 2 h and then for another 20-24 h at 20 °C. Cells were harvested at 4,500g and 4 °C for 15 min, the pellets were resuspended in equilibration buffer (50 mM TRIS-HCl, 300 mM NaCl, pH 8.8) and then cell disruption was carried out by ultrasonication (Bandelin MS 73, Berlin, Germany; 2 × 5 min) on ice. The cell debris were removed by centrifugation at 15,000g and 4 °C for 45 min and the supernatant was filtered using a 0,2 µm bottle top filter.

The purification was carried out as described by Dreßen *et al.* (DOI: 10.1016/j.jbiotec.2017.04.005) with an ÄKTA Start (Cytiva, USA) and a HisTrap FF Crude column (1 mL, Cytiva, USA). For storage of the purified PAL, the buffer was exchanged to a 2 mM TRIS-HCl buffer (pH 8.8) with PD-10 desalting columns (Cytiva, USA) and dried for 3 h at 5 mbar using a RVC 2-33 IR rotary vacuum concentrator (Christ, Germany). The protein concentration was determined with a bicinchoninic acid assay (Thermo Scientific, USA). The presence of the PAL in all steps was checked by sodium dodecyl sulfate polyacrylamide gel electrophoresis (SDS-PAGE) as done previously*.*^[10]^

## 3.16 Deamination of L-Phenylalanine with PAL to test enzyme activity at different pH

A substrate solution containing 30 mM of L-phenylalanine in 50 mM TRIS-HCl buffer at pH 8.8 was prepared. 982 μL of substrate solution was filled into 1.5 mL QS-cuvettes with caps, and remained for 6 min at 30°C. The reaction was started by adding 32 μL of the enzyme solution (0.525 mg/mL) and inverting the cuvette 3 times. Production of transcinnamic acid was followed at 275 nm for 2 min. The activity was calculated using the slope of the first 80 s and following equation 1 was used:

$specific activity\left[ \frac{U}{mg} \right]= \frac{\frac{{\Delta A}_{275nm}}{min}*V*f}{\varepsilon_{275nm}*d*c*v}$ Equation 1

∆A_275nm_/min = increase of absorbance at 275 nm per minute [min^-1^]

V = total assay volume

f = dilution factor of the enzyme solution

ε_275nm_ = extinction coefficient of trans-cinnamic acid [17.218 mM^-1^cm^-1^]

d = path length cuvette

c= enzyme concentration [mg/mL]

v = volume enzyme sample in the same measurement, 30 mM of L-phenylalanine was dissolved in 50 mM TRIS-HCl buffers with different pH values (pH 5-8) to test the enzyme activity at other pH.

## 3.17 FACS analysis

FACS characterization was carried out using a Cytoflex. At least 10000 particles were characterized to determine the 2D dot plots of the FSC and SSC light. The fluorescence signals of the individual particles were characterized by a 565 nm laser. Sample characterizations for FACS were as following: (i) 130 μL of coacervates (volume ratio is defined under coacervate formation) was characterized to determine the FACS signal for an individual population of coacervate droplets; (ii) 130 μL of Coa@DMPC (volume ratio is defined under coacervate formation under FITC and phospholipid staining) was characterized to determine the FACS signal for an individual population of Coa@DMPC. (iii) an aqueous mixture containing after 15 min of incubation with 100 μL Coa@DMPC and 50 μL RhB-Azo-Psomes (15wt%) was characterized to determine the FACS signal for an individual population of mixed solution.

## 3.18 Study on GOx enzyme activity loaded by Coa@DMPC

20 mg/mL of Su-Am stock solution was diluted to 1 mg/mL and 5 mg/mL of PMEDAB-Chol stock solution was diluted to 2 mg/mL. The coacervate droplets were prepared by mixing the polyelectrolyte solutions by volume ratio of 1:1. The final solution volume of coacervate droplets was 400 μL. To this coacervate droplet solution 50 μL of GOx (1 mg/mL H_2_O) was added to load it inside coacervate. Then 37.5 μL of phospholipid solution (20 mg/mL) was added and kept it for 20 min without shaking to form GOx-loaded Coa@DMPC. The resulting enzymatic-active coacervate droplet solution was then diluted with 100 µL of H_2_O to make the pH change obviously. To check the acidic pH changes in GOx-loaded Coa@DMPC solutions 50 μL of glucose in different concentrations (0.7 mg/mL, 1.3 mg/mL and 1.5mg/mL) was added. The pH changes were recorded over time by a pH meter (**Figure 4b**).

## 3.19 Study on PAL enzyme activity loaded by Coa@DMPC

20 mg/mL of Su-Am stock solution was diluted to 1 mg/mL and 5 mg/mL of PMEDAB-Chol stock solution was diluted to 2 mg/mL. The coacervate droplets were prepared by mixing the polyelectrolyte solutions by volume ratio of 1:1. The final solution volume of coacervate droplets was 400 μL. To this coacervate droplet solution 50 μL of PAL (2.1 mg/mL, H_2_O) was added to load it inside coacervate droplets. Then 37.5 μL of phospholipid solution (20 mg/mL) was added and kept it for 20 min without shaking to form PAL-loaded Coa@DMPC. The resulting enzymatic-active coacervate droplet solution was then diluted with 100 µL of H_2_O to make the pH change obviously. To check the basic pH changes in PAL-loaded Coa@DMPC solutions, 50 μL of L-phenylalanine in different concentrations (0.9 mg/mL, 1.2 mg/mL and 1.5 mg/mL) was added. The pH changes were recorded over time by a pH meter (**Figure 4c**).

## 3.20 Study on the fluorescence changes of FITC in Coa@DMPC loaded with GOx after adding glucose

20 mg/mL of Su-Am stock solution was diluted to 1 mg/mL and 5 mg/mL of PMEDAB-Chol stock solution was diluted to 2 mg/mL. The coacervate droplets were prepared by mixing the polyelectrolyte solutions by volume ratio of 1:1. The final solution volume of coacervate droplets was 80 μL. To this coacervate droplet solution 50 μL of GOx (1 mg/mL, H_2_O) and 5 μL of FITC (1 mg/mL, H_2_O) were added to load it inside coacervate droplets. Then 9.6 μL of phospholipid solution (20 mg/mL) was added and kept it for 20 min without shaking to form GOx-loaded Coa@DMPC. Then, 10 μL of GOx-loaded COa@DMPC solution was taken and 1 μL of glucose (1.5 mg/mL) was added to observe the fluorescence change of FITC very quickly (**Figure 4d**).

## 3.21 Study on the docking of Azo-Psomes with Coa@DMPC by CLSM

20 mg/mL of Su-Am stock solution was diluted to 1 mg/mL and 5 mg/mL of PMEDAB-Chol stock solution was diluted to 2 mg/mL. The coacervate droplets were prepared by mixing the polyelectrolyte solutions by volume ratio of 1:1. The final solution volume of coacervate droplets was 400 μL. To this coacervate droplet solution 50 μL of Cy5-GOx (1 mg/mL, H_2_O) and 50 μL of Cy7-PAL (2.1 mg/mL, H_2_O) were added to load it inside coacervate. Then 37.5 μL of phospholipid solution (20 mg/mL, 5% NBD-PC) was added and kept it for 20 min without shaking to form Cy5-GOx/Cy7-PAL-loaded Coa@DMPC. 10 µL of the above solution was observed by CLSM to observe the Cy5-GOx/Cy7-PAL-loaded Coa@DMPC (**Figure S21**).

Then 200 μL Cy5-Azo-Psomes (15wt%) (1 mg/mL) was added to dock on Coa@DMPC surface. After 20 min of equilibrium (Waiting for docking of Azo-Psomes procees), 10 µL of the above solution was observed by CLSM to observe the docking of HMC (**Figures 5a-b**).

## 3.22 Study on the one-time release of L-phenylalanine in Azo-Psomes

200 μL of Azo-Psomes (15 wt%) (1 mg/mL) loaded with L-phenylalanine was added to 300 μL of H_2_O, the pH was adjusted to 6.5 with 0.5 M HCl, and 50 μL of PAL (2.1 mg/mL) was added, the change of pH was observed by pH meter. When the pH rose to a stable level, quickly adjusted the pH to around 6.5 with 0.5 M HCl and continued to observe the changes of pH (**Figure S24**).

## 3.23 Study on the glucose-triggered enzymatic reaction between Coa@DMPC and Azo-Psomes

20 mg/mL of Su-Am stock solution was diluted to 1 mg/mL and 5 mg/mL of PMEDAB-Chol stock solution was diluted to 2 mg/mL. The coacervate droplets were prepared by mixing the polyelectrolyte solutions by volume ratio of 1:1. The final solution volume of coacervate droplets was 400 μL. To this coacervate droplet solution 50 μL of GOx (1 mg/mL, H_2_O) and 50 μL of PAL (2.1 mg/mL, H_2_O) were added to load it inside coacervate. Then 37.5 μL of phospholipid solution (20 mg/mL) was added and kept it for 20 min without shaking to form GOx/PAL-loaded Coa@DMPC. 100 μL of H_2_O was added to dilute the buffer to make the pH change obviously. Then 200 μL of Azo-Psomes (15 wt%) (1 mg/mL) loaded with L-phenylalanine was added to dock on Coa@DMPC surface. After 20 min of equilibrium (Waiting for docking of Azo-Psomes) the pH was adjusted to 7.5, 70 μL of glucose (1.5 mg/mL) was added, and the change of pH was observed by pH meter (**Figure 5c**).

## 3.24 Study on the glucose-triggered enzymatic reaction between coacervate droplets and Azo-Psomes

20 mg/mL of Su-Am stock solution was diluted to 1 mg/mL and 5 mg/mL of PMEDAB-Chol stock solution was diluted to 2 mg/mL. The coacervate droplets were prepared by mixing the polyelectrolyte solutions by volume ratio of 1:1. The final solution volume of coacervate droplets was 400 μL. To this coacervate droplet solution 50 μL of GOx (1 mg/mL, H_2_O) and 50 μL of PAL (2.1 mg/mL, H_2_O) were added to load it inside coacervate. Kept it for 20 min without shaking to form GOx/PAL-loaded coacervate. 100 μL of H_2_O was added to dilute the buffer to make the pH change obviously. Then 200 μL of Azo-Psomes (15 wt%) (1 mg/mL) loaded with L-phenylalanine was added. After 20 min the pH was adjusted to 7.5, 70 μL of glucose (1.5 mg/mL) was added, and the change of pH was observed by pH meter (**Figure 5c**).

## 3.25 Study on the docking of Azo-Psomes on Coa@DMPC surface under pH cycle changes

20 mg/mL of Su-Am stock solution was diluted to 1 mg/mL, and 5 mg/mL of PMEDAB- Chol stock solution was diluted to 2 mg/mL. The coacervate droplets were prepared by mixing the polyelectrolyte solutions by volume ratio of 1:1. The final solution volume was 400 μL. To this coacervate droplet solution 50 μL of Cy5-GOx (1 mg/mL, H_2_O) and 50 μL of PAL (2.1 mg/mL, H_2_O) were added to load it inside coacervate. Then 37.5 μL of phospholipid solution (20 mg/mL) was added and kept it for 20 min without shaking to form Cy5-GOx/PAL-loaded Coa@DMPC. 100 μL of H_2_O was added to dilute the buffer to make the pH change obviously. Then, 200 μL of Azo-Psomes (15 wt%) (1 mg/mL) was added to dock on Coa@DMPC surface. After 20 min of equilibrium (Waiting for docking of Azo-Psomes procees), the pH was adjusted to 7.5, 70 μL of glucose (1.5 mg/mL) was added and the change of pH was observed by pH meter. After pH reduction, 80 μL of L-phenylalanine (1.5 mg/mL) was added and the change of pH was observed by pH meter again. Cyclic enzyme-induced pH-switches were explored by repeated addition of glucose and L-phenylalanine for three times (**Figure** **5e**). After each pH change, 10 μL of the solution was extracted to observe the docking of HMC by CLSM (**Figure 5d**).

## 3.26 Study on the docking of Azo-Psomes on Coa@DMPC surface after pH reduction

20 mg/mL of Su-Am stock solution was diluted to 1 mg/mL and 5 mg/mL of PMEDAB-Chol stock solution was diluted to 2 mg/mL. The coacervate droplets were prepared by mixing the polyelectrolyte solutions by volume ratio of 1:1. The final solution volume of coacervate droplets was 400 μL. To this coacervate droplet solution 50 μL of Cy5-GOx (1 mg/mL, H_2_O) was added to load it inside coacervate. Then 37.5 μL of phospholipid solution (20 mg/mL) was added and kept it for 20 min without shaking to form Cy5-GOx-loaded Coa@DMPC. 100 μL of H_2_O was added to dilute the buffer to make the pH change obviously. Then, 200 μL of RhB-Azo-Psomes (15 wt%) (1 mg/mL) was added to dock on Coa@DMPC surface. After 20 min of equilibrium (Waiting for docking of RhB-Azo-Psomes procees) the pH was adjusted to 7.5, then 70 μL of glucose (1.5 mg/mL) was added, and the change of pH was observed by pH meter. Then, the docking of RhB-Azo-Psomes on Coa@DMPC surface was observed by CLSM at different time points.

## 3.27 TMB assay at different pH values of (βCD)_2_Hemin-Azo-Psomes

Sample 1 at pH 6: 2.8 mL of 1 mM PBS at pH 6 and 200 μL of (βCD)_2_Hemin-Azo-Psomes stock solution (0.5 mg/mL) were mixed. Sample 2 at pH 7.4: 2.8 mL of 1 mM PBS at pH 7.4 and 200 μL of (βCD)_2_Hemin stock solution (0.5 mg/mL) were mixed.

TMB assay: 200 μL of the prepared samples, 3 μL of H_2_O_2_ (0.6 M) and 4 μL of TMB (50 mM) were mixed and monitored at 656 nm by UV-VIS measurements for 10 min at 25°C. All samples were measured in triplicate.

## 3.28 Regulation of peroxidase-mimicking activity of (βCD)_2_Hemin-Azo-Psomes

20 mg/mL of Su-Am stock solution was diluted to 1 mg/mL and 5mg/mL of PMEDAB- Chol stock solution was diluted to 2 mg/mL. The coacervate droplets were prepared by mixing the polyelectrolyte solutions by volume ratio of 1:1. The final solution volume was 400 μL. To this coacervate droplet solution 50 μL of GOx (1 mg/mL, H_2_O) and 50 μL of PAL (2.1 mg/mL, H_2_O) were added to load it inside coacervate. Then 37.5 μL of phospholipid solution (20 mg/mL) was added and kept it for 20 min without shaking to form GOx/PAL-loaded Coa@DMPC. Then 200 μL of (βCD)_2_Hemin-Azo-Psomes (15 wt%) (1 mg/mL) was added to dock on the Coa@DMPC surface. Then 100 μL of H_2_O was added to dilute the buffer to make the pH change obviously, and the pH of HMC solution was adjusted pH to 7.4. From this stock solution different samples can be taken for spatio-temporal enzymatic(-like) reaction of HMC (**Figure 6**).

Then, 150 μL of the stock solution and 1 μL of TMB (50 mM) were mixed at 25°C. Subsequently, glucose 15 μL (2 mg/mL) or L-Phenylalanine 20 μL (2 mg/mL) were added cyclically and UV-VIS measurements, carried out at wavelength 656 nm, were taken at different time points (**Figure 6e**). Glucose was only added stepwise in a control experiment (**Figure 6e**).

# 4. Additional Schemes, Figures and Tables

**
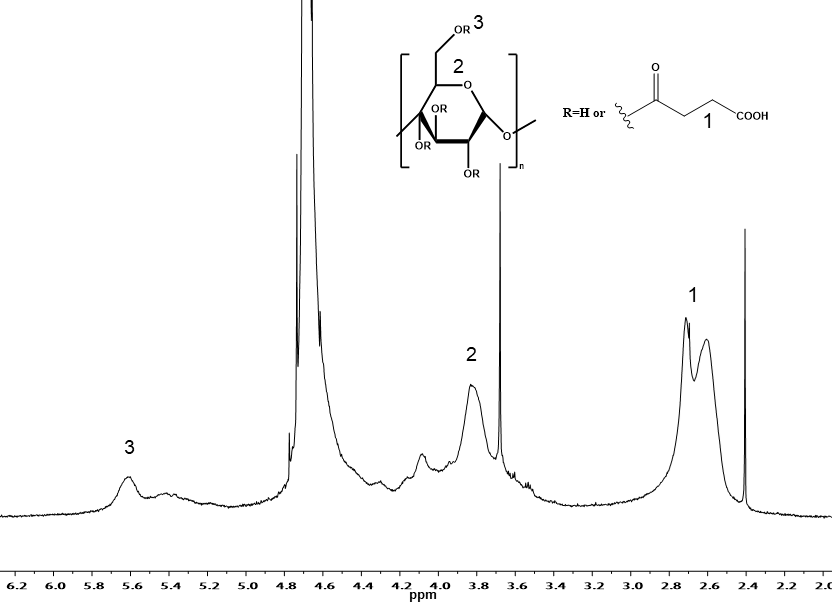
**

**Figure S2.** ^1^H NMR (500 MHz, D_2_O) of Su-Am.


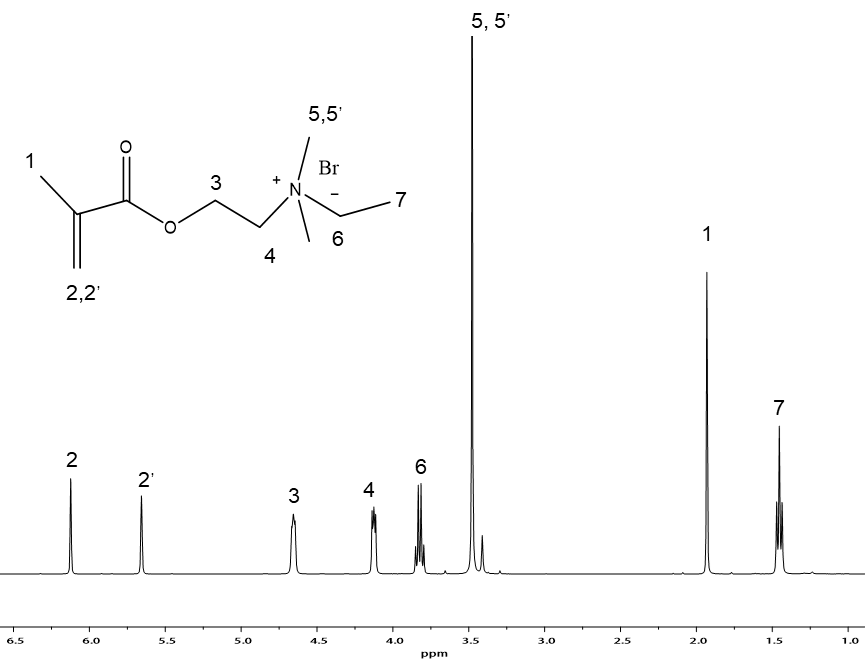


**Figure S3.** ^1^H NMR (500 MHz, D_2_O) of methacryloxyethyl dimethylethanyl ammonium bromide (MEDAB).


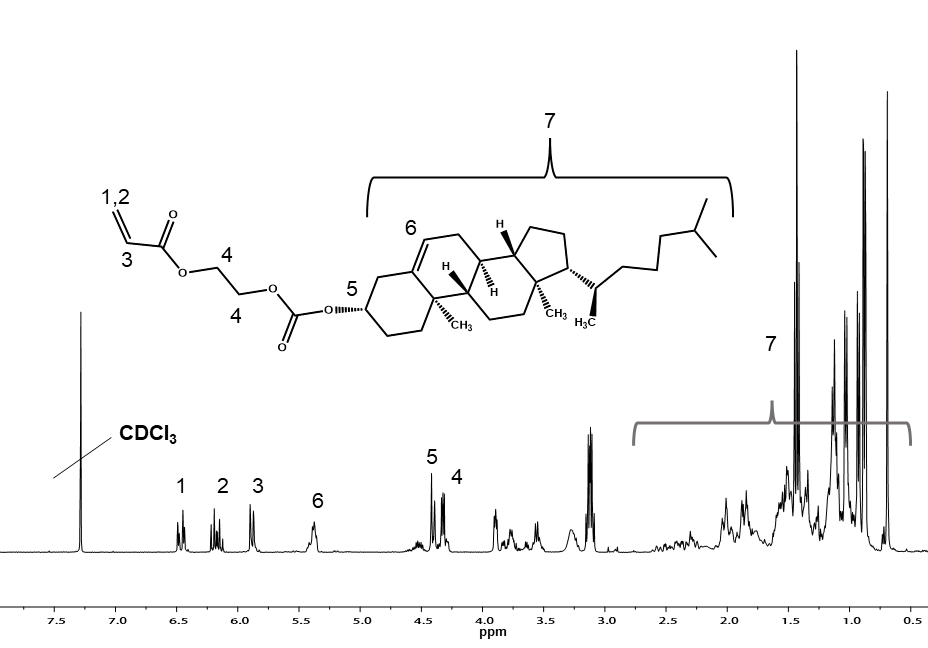


**Figure S4.** ^1^H NMR (500 MHz, CDCl_3_) of cholesteryl acryloyoxy ethyl carbonate.


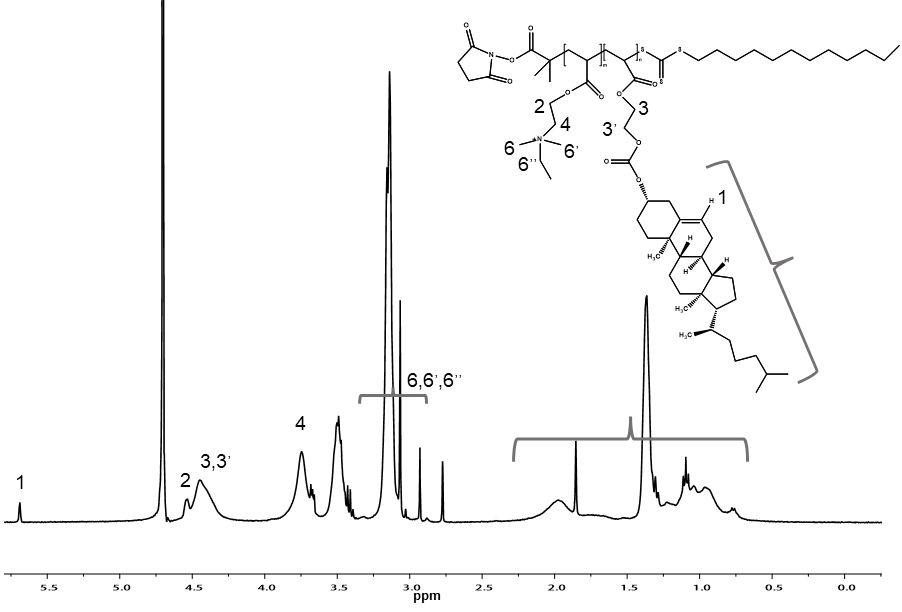


**Figure S5.** ^1^H NMR (500 MHz, D_2_O) of PMEDAB-Chol.

**Scheme S1.** Schematic presentation of synthesized block copolymers, BCP A, Azo-BCP, and RhB-BCP A, via ATRP and click reaction conditions. Further details of the composition and molecular weight presented in **Table S1**. DEAEMA = 2-(*N,N*-diethylamino)ethyl methacrylate. DMIBMA = photo-crosslinker 4-(3,4-dimethylmaleimido)butyl methacrylate (DMIBMA). Azo-PP = propargyl-modified azobenzene. RhB-MA = rhodamine B-conjugated methacrylate.

**Scheme S2.** Schematic presentation for the synthesis of Cy5-labeled BCP A (Cy5-BCP A) to finally fabricate Cy5-labeled Azo-Psomes (Cy5-Azo-Psomes). Further details of the composition and molecular weight for PDSMA-BCP A are presented in **Table S1**. DEAEMA = 2-(*N,N*-diethylamino)ethyl methacrylate. DMIBMA = photo-crosslinker 4-(3,4-dimethylmaleimido)butyl methacrylate (DMIBMA). PDSMA = 2-(pyridyl-2-disulfide)ethyl methacrylate.

| Azo-BCP | N3-PEG79-Br  eq. [mol] [mg] | DEAEMA  eq. [mol] [mL] | DMIBMA  eq. [mol] [mg] | Azo-PP  eq. [mol] [mg] | 2,2´-Bipyridyl  eq. [mol] [mg] | CuBr  eq. [mol] [mg] | EMK  [mL] |
| --- | --- | --- | --- | --- | --- | --- | --- |
| AS331 | 1  2,793*10^-5^  100 | 88  2,458*10^-3^  0,494 | 22  0,615*10^-3^  163,0 | 1,2  3,35*10^-5^  7,92 | 2  5,59*10^-5^  8,7 | 1  2,793*10^-5^  4,0 | 5 |
| RhB-BCP A | **PEG45-Br**  eq. [mol] [mg] | **DEAEMA**  eq. [mol] [mL] | **DMIBMA**  eq. [mol] [mg] | **RhB-MA**  eq. [mol] [mg] | **2,2´-Bipyridyl**  eq. [mol] [mg] | **CuBr**  eq. [mol] [mg] | **EMK:DMF (1:1)**  [mL] |
| AS330 | 1  4,653*10^-5^  100 | 88  4,095*10^-3^  0,823 | 22  1,024*10^-3^  271,6,0 | 1  4,653*10^-5^  31,0 | 2  9,306*10^-5^  14,5 | 1  4,653*10^-5^  6,7 | 4 |
| PDSMA-BCP A | **PEG45-RAFT**  eq. [mol] [mg] | **DEAEMA**  eq. [mol] [mL] | **DMIBMA**  eq. [mol] [mg] | **PDSMA**  eq. [mol] [mg] | **AIBN**  eq. [mol] [mg] |  | **1,4-Dioxane**  [mL] |
| AS314 | 1  4,261*10^-5^  100 | 67,5  2,877*10^-3^  0,578 | 18  0,767*10^-3^  203,5 | 4,5  0,192*10^-3^  48,9 | 0,5  2,13*10^-5^  3,5 |  | 4 |
| BCP A | **PEG45-Br**  eq. [mol] [mg] | **DEAEMA**  eq. [mol] [mL] | **DMIBMA**  eq. [mol] [mg] |  | **2,2´-Bipyridyl**  eq. [mol] [mg] | **CuBr**  eq. [mol] [mg] | **EMK**  [mL] |
| AS278 | 1  4,653*10^-5^  100 | 88  4,095*10^-3^  0,823 | 22  1,024*10^-3^  271,6,0 |  | 2  9,306*10^-5^  14,5 | 1  4,653*10^-5^  6,7 | 4 |
| ^a^ EMK = ethylmethyl ketone; DMF = dimethylformamide. | | | | | | | |

**Table S1.** Used educts for carrying out ATRP and RAFT to synthesize various block copolymers.

**Table S2.** Specification of synthesized block copolymers^a^

| **Block copolymer** | **Composition^c^** | **Block ratio** | **M_n_^b^**  g/moL^-1^ | **M_w_^b^**  g/moL^-1^ | **Ð^b^**  M_w_/M_n_ | **M_n_^c^**  g/moL^-1^ |
| --- | --- | --- | --- | --- | --- | --- |
| Azo-BCP  AS 331 | Azo-benzene-PEG_79_-*b*-(PDEAEMA_97_-*co*-DMIBMA_27_)  *94% conjugation of azo-benzene* | 1:1.57 | 35,450 | 40,500 | 1.14 | 28,950 |
| BCP A  AS 371 | PEG_45_-*b*-(PDEAEMA_93_-*co*-DMIBMA_24_) | 1:2.6 | 36,850 | 42,300 | 1.15 | 24,100 |
| RhB-BCP A  AS 330 | PEG_45_-*b*-(PDEAEMA_94_-*co*-DMIBMA_25_-*co*-RhB-MA_0.14_) | 1:2.6 | 45,950 | 59,550 | 1.35 | 26,600 |
| PDSMA-BCP A  AS 314 | PEG_45_-*b*-(PDEAEMA_68_-*co*-DMIBMA_21_-*co*-PDSMA_4_) | 1:2.1 | 33,450 | 58,150 | 1.74 | 21,340 |
| ^a^Block ratio is given by the ratio of the hydrophilic block segment and the hydrophobic block segment, results overview about differently determined molar masses and Đ. ^b^Determined by SEC. ^c^Calculated by ^1^H-NMR. | | | | | | |


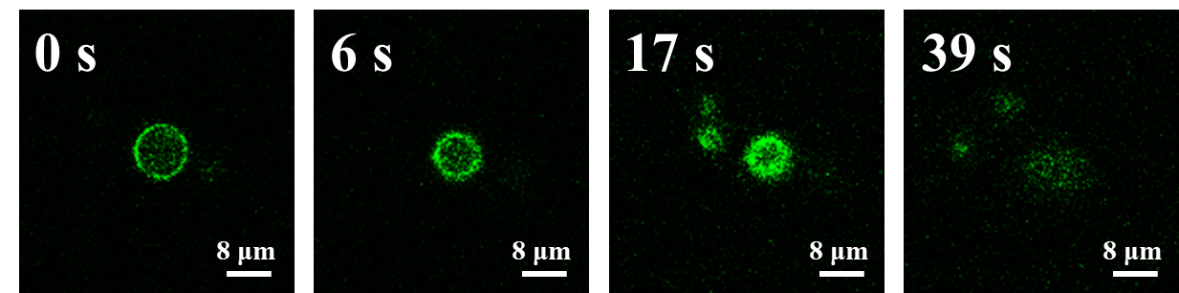


**Figure S6.** Time-dependent corresponding confocal fluorescence images of morphological changes in membranized Coa@DMPC, visualized by NBD-PD as dye-labeled phospholipid, through the addition of β-CD to disrupt the molecular interactions of phospholipids and cholesterol within cholesterol-stabilized phospholipid membrane of Coa@DMPC within cholesterol-stabilized phospholipid membrane of Coa@DMPC

**Figure S7.** Zeta potential measurements for Su-Am (blue), PMEDAB-Chol (green), coacervate (grey) and Coa@DMPC (red) at room temperature. From the graph, it can be inferred that coacervate and Coa@DMPC were positively charged.

**Figure S8.** ^1^H NMR (500 MHz, CDCl_3_) of BCP A.

__

**Figure S9.** ^1^H NMR (500 MHz, CDCl_3_) of Azo-BCP.

**Figure S10.** ^1^H NMR (500 MHz, CDCl_3_) of RhB-BCP A.

**
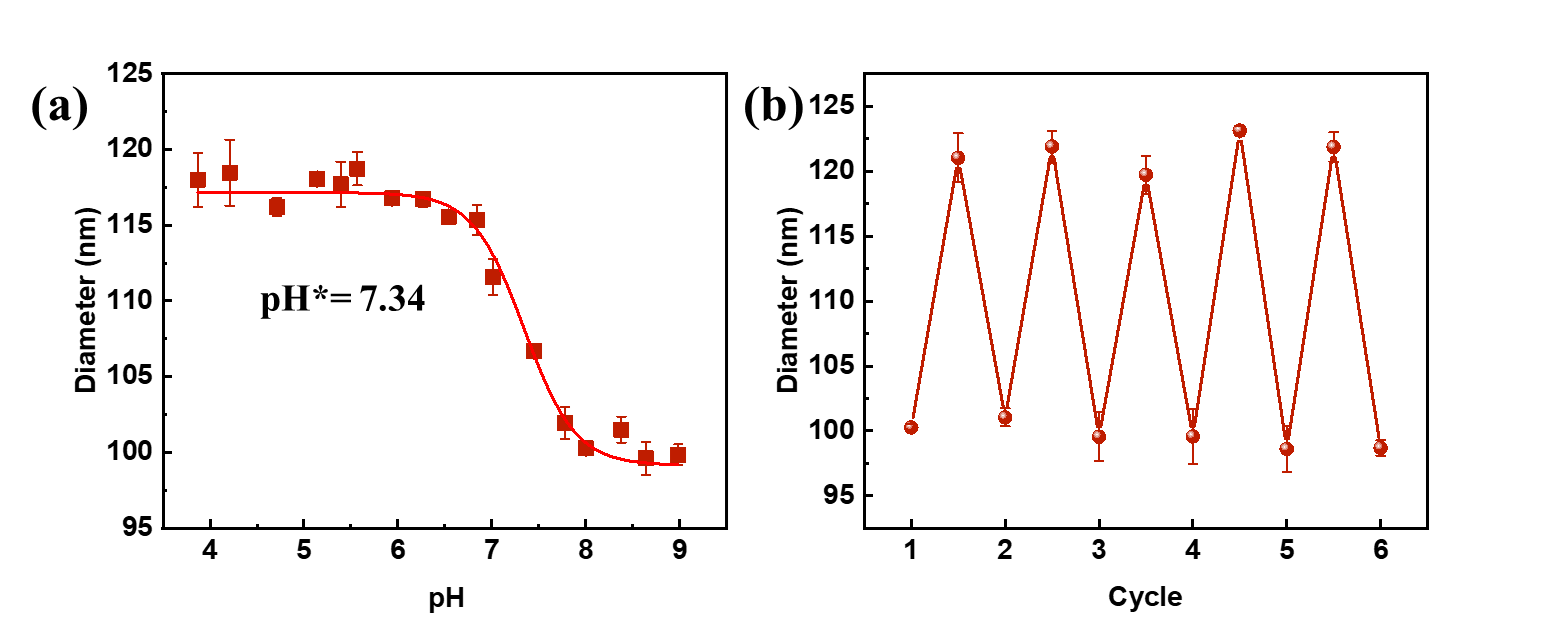
Figure S11.** (a) pH-dependent DLS titration curves of RhB-Azo-Psomes (15wt%, red) for determining the pH*. (b) Cyclic pH-switches of RhB-Azo-Psomes at pH 4 and pH 8 determined by DLS.

The results show that Rhodamine B labeling of Azo-Psomes does not alter their pH responsiveness, leading to good balance of swelling to shrinking behavior.


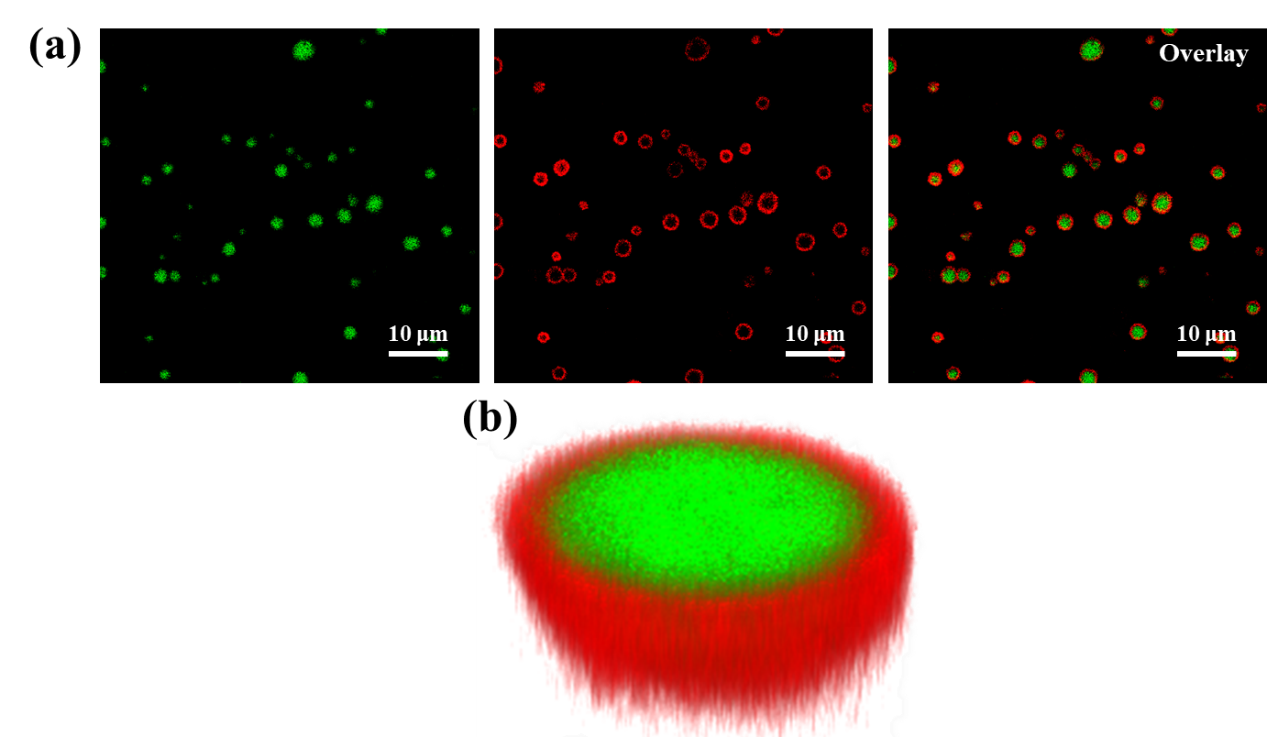


**Figure S12. (**a, b) Confocal microscopy images of RhB-Azo-Psomes (25wt%, red), showing the expected docking on the surface of FITC-Coa@DMPC (green) via the fluorescence colocalization analysis and 3D confocal image in (b). The volume ratio of Azo-Psomes (25wt%) to FITC-Coa@DMPC is 2:1. The docking on the surface of FITC-Coa@DMPC is much denser compared to RhB-Azo-Psomes (15 wt%) (**Figure 3c**).


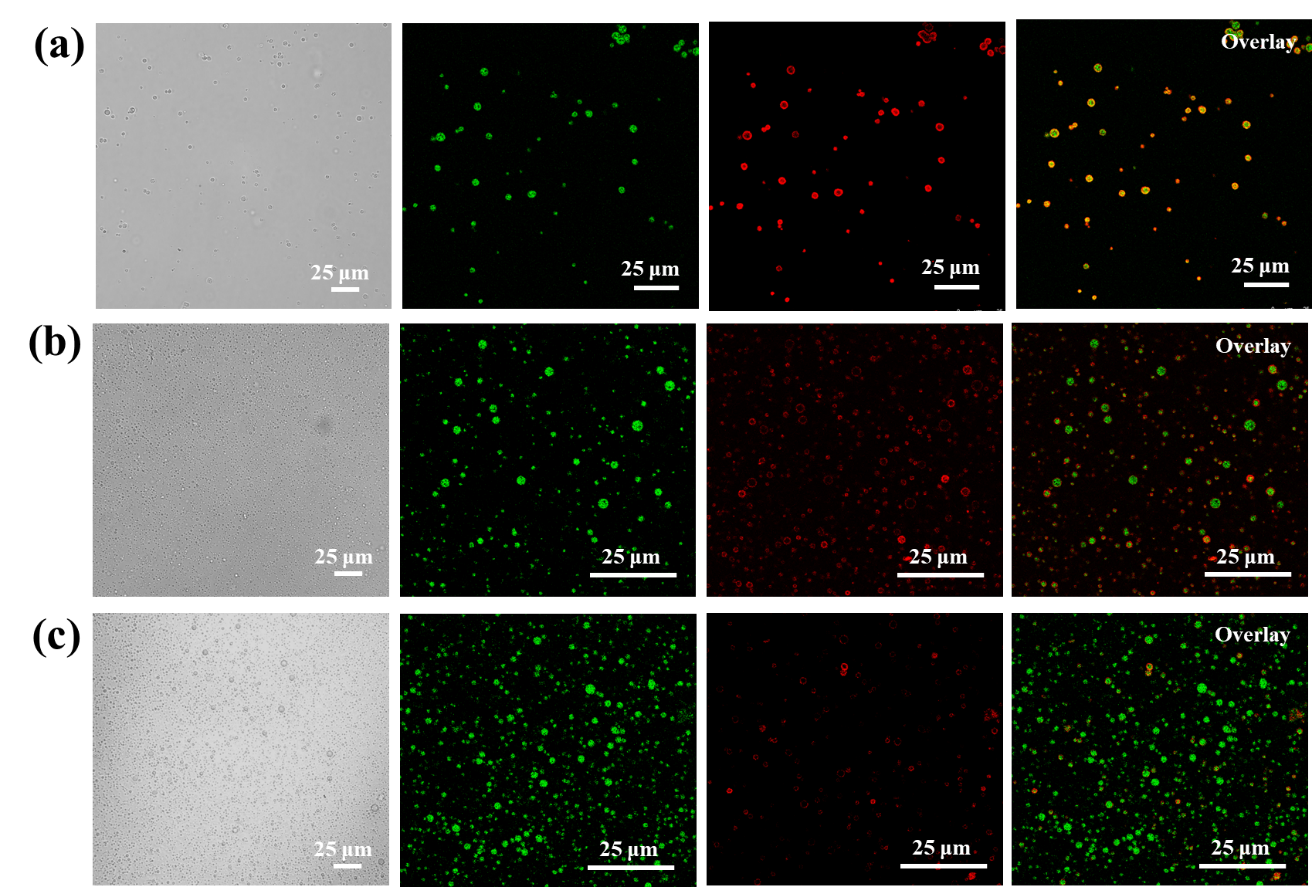


**Figure S13.** The docking of RhB-Azo-psomes (15 wt%, red) on FITC-Coa@DMPC surface (green) with different volume ratios was observed by CLSM. The corresponding volume ratios of FITC-Coa@DMPC and RhB-Azo-Psomes (15 wt%) in (a), (b) and (c) were 1:1, 2:1 and 10:1.

The results show that the docking density of RhB-Azo-Psomes (15 wt%) on FITC-Coa@DMPC surface decreases as the number of RhB-Azo-Psomes (15 wt%) is reduced.

**
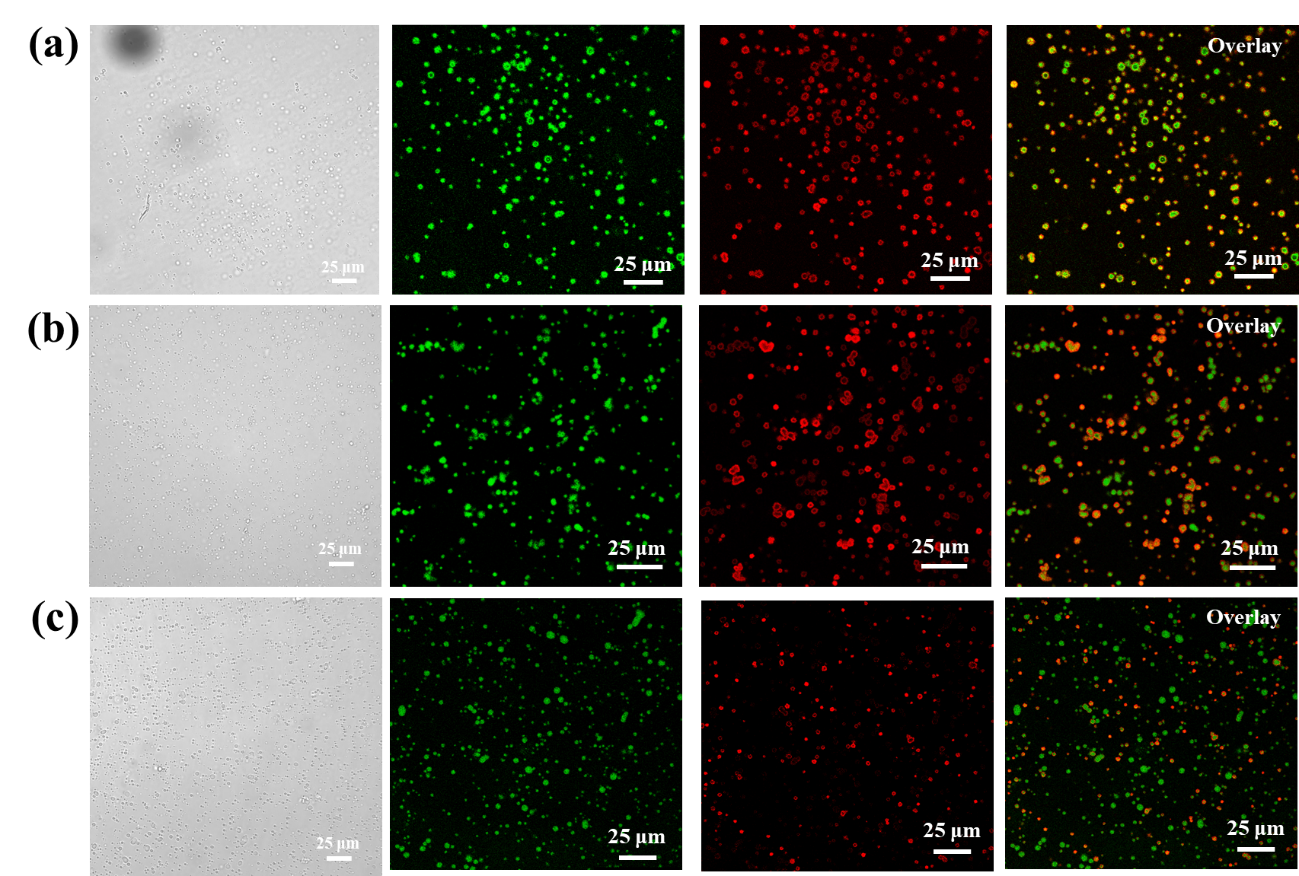
Figure S14.** The docking of RhB-Azo-Psomes (25 wt%, red) on FITC-Coa@DMPC surface (green) with different volume ratios is observed by CLSM. The corresponding volume ratios of FITC-Coa@DMPC and RhB-Azo-Psomes (25 wt%) in (a), (b) and (c) were 1:1, 2:1 and 10:1.

The results show that the docking density of RhB-Azo-Psomes (25wt%) on FITC-Coa@DMPC surface decreases as the number of RhB-Azo-Psomes (25 wt%) is reduced. However, with the same volume ratio, the docking density of RhB-Azo-Psomes (25 wt%) on FITC-Coa@DMPC surface is higher than that of RhB-Azo-Psomes (15 wt%) based HMC (**Figure S13**).

**
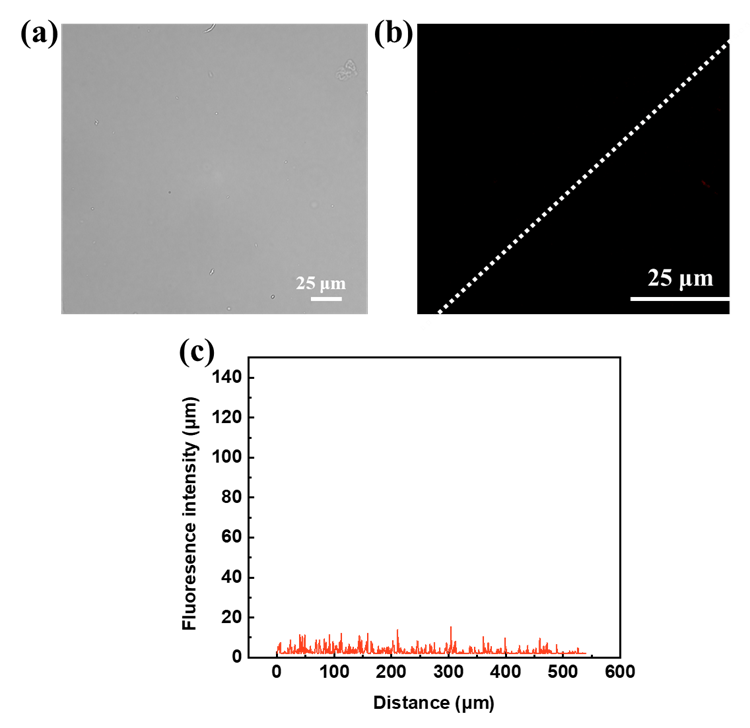
**

**Figure S15.** (a, b) Optical (a) and confocal (b) microscopy images of RhB-Azo-Psomes (15 wt%, red). (c) Corresponding fluorescence intensity analysis using (position of imaginary line is shown in (b). RhB-Azo-Psomes (15 wt%) cannot be directly observed by CLSM due to their small size.

**
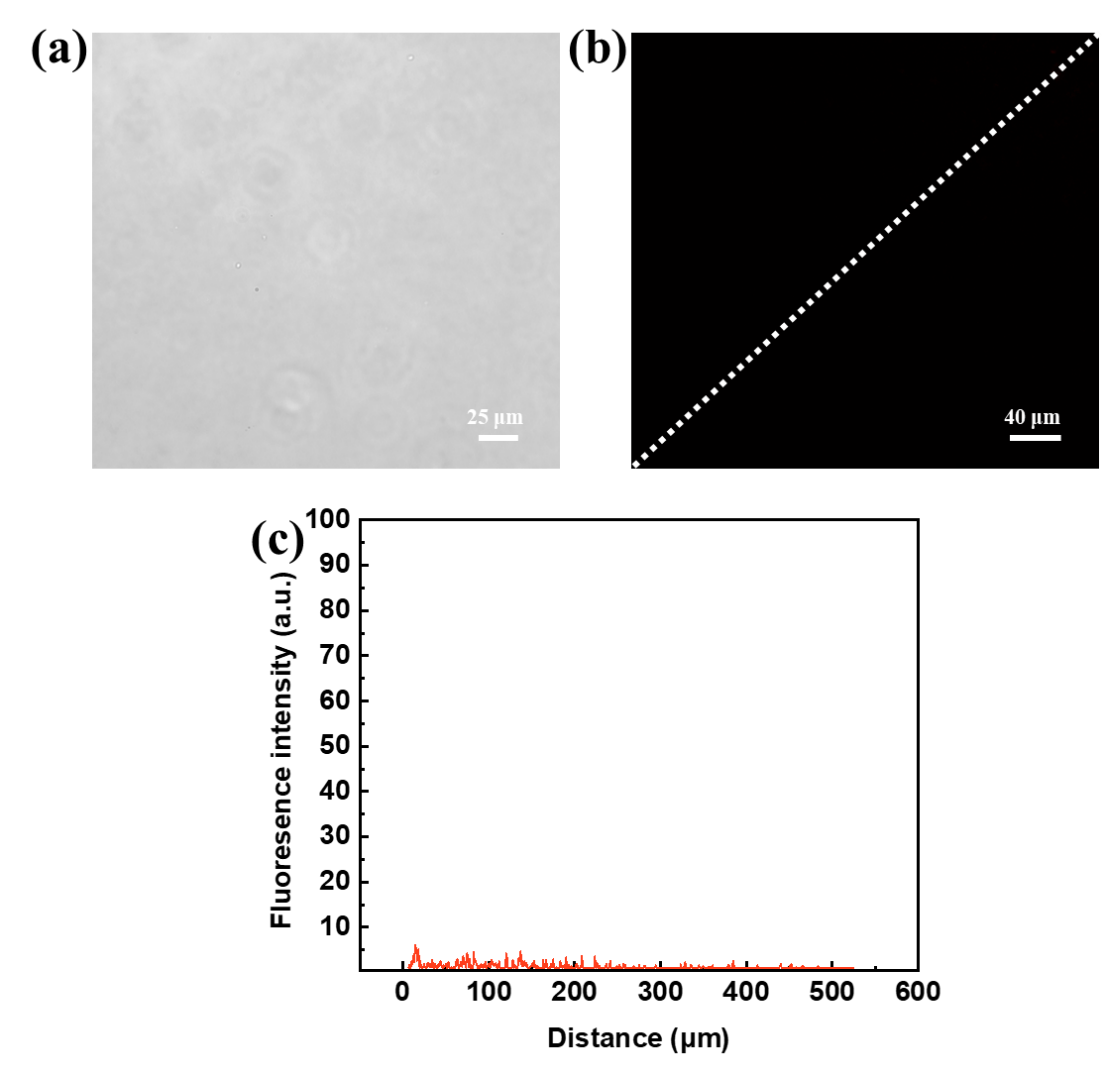
Figure S16.** (a, b) Optical (a) and confocal (b) microscopy images of RhB-Azo-Psomes (25 wt%, red). (c) Corresponding fluorescence intensity analysis using (position of imaginary line is shown in (b). RhB-Azo-Psomes (25 wt%) cannot be directly observed by CLSM due to their small size.


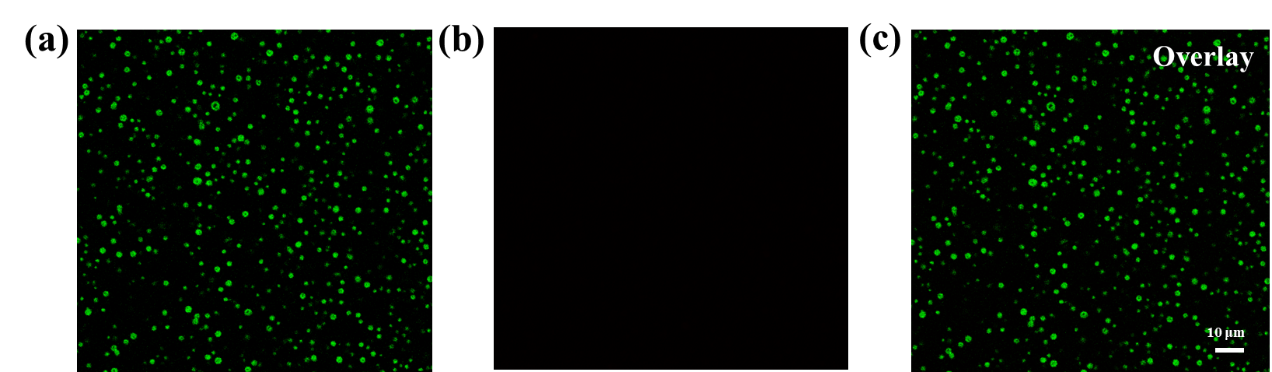


**Figure S17.** (a, b and c) CLSM images of FITC-coacervates (without DMPC, green) and RhB-Azo-Psomes (25 wt%, red).

RhB-Azo-Psomes (25 wt%) do not dock to FITC-coacervate droplets or are not captured by FITC-coacervate droplets. There is no insertion site for azobenzene in the absence of phospholipid bilayers. Furthermore, the two compartments do not undergo preferred interactions because of their existing electrostatic repulsion forces (cationic zeta potential of coacervate in **Figure S7**; e.g. cationic zeta potential of ferrocene-modified Psomes at neutral pH^[1a]^).

**
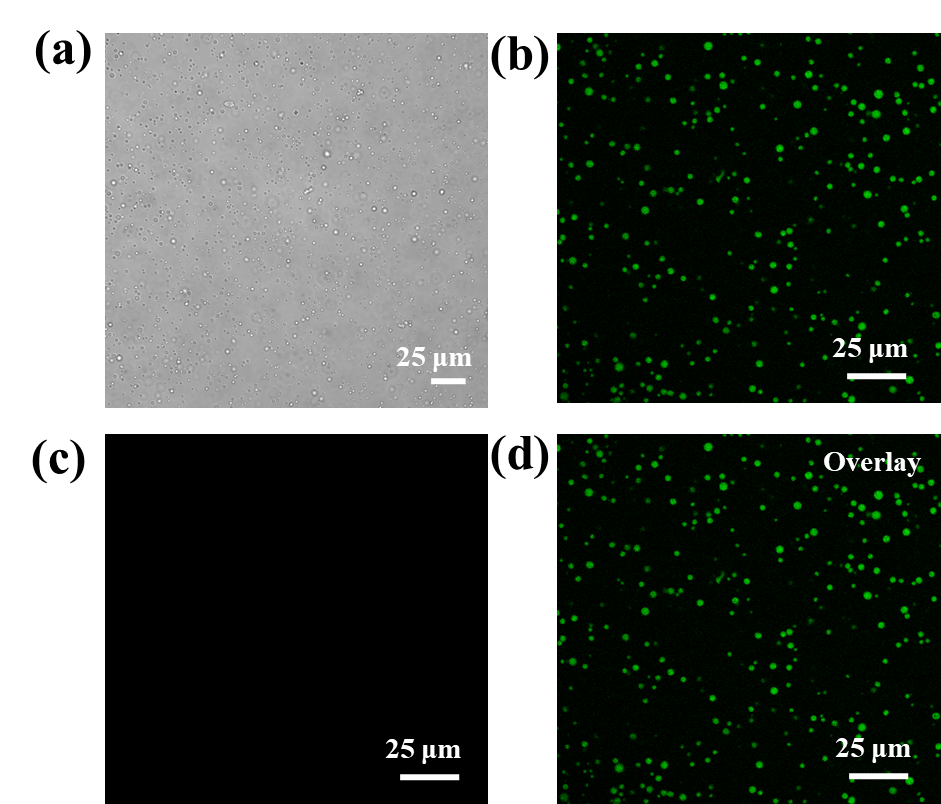
Figure S18.** (a, b, c and d) CLSM images of FITC-Coa@DMPC (green) and RhB-Psomes A (red).

Due to the absence of azobenzene in RhB-Psomes A, RhB-Psomes A are also unable to dock on the FITC-Coa@DMPC surface. Furthermore, this is further supported by the presence of their existing cationic repulsion forces (cationic zeta potential of coacervate in **Figure S7**; e.g. cationic zeta potential of ferrocene-modified Psomes at neutral pH^[1a]^).


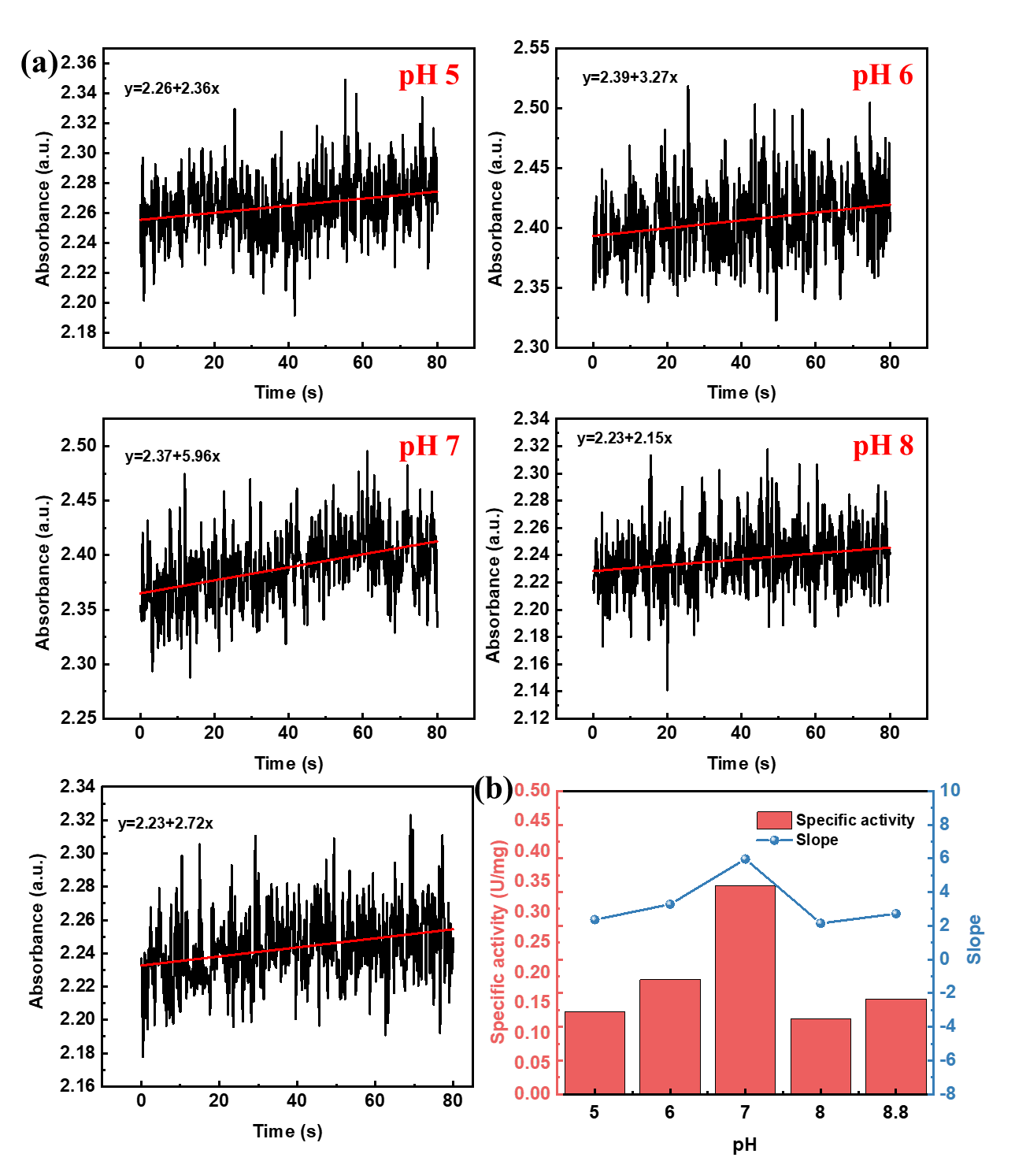


**Figure S19.** Deamination of L-Phenylalanine with PAL to test the enzyme activity of PAL at different pH values. (a) The absorbance changes at 275 nm for the first 80 s were measured by deamination of L-Phenylalanine with PAL at different pH. (b) Statistical histogram of enzyme activity of PAL at different pH based on Equation 1.

This activity test is necessary to know the PAL functions at acidic pH for inducing requested pH jumps from acidic to basic environment in final experiments (e.g. presented results in **Figure 5**).


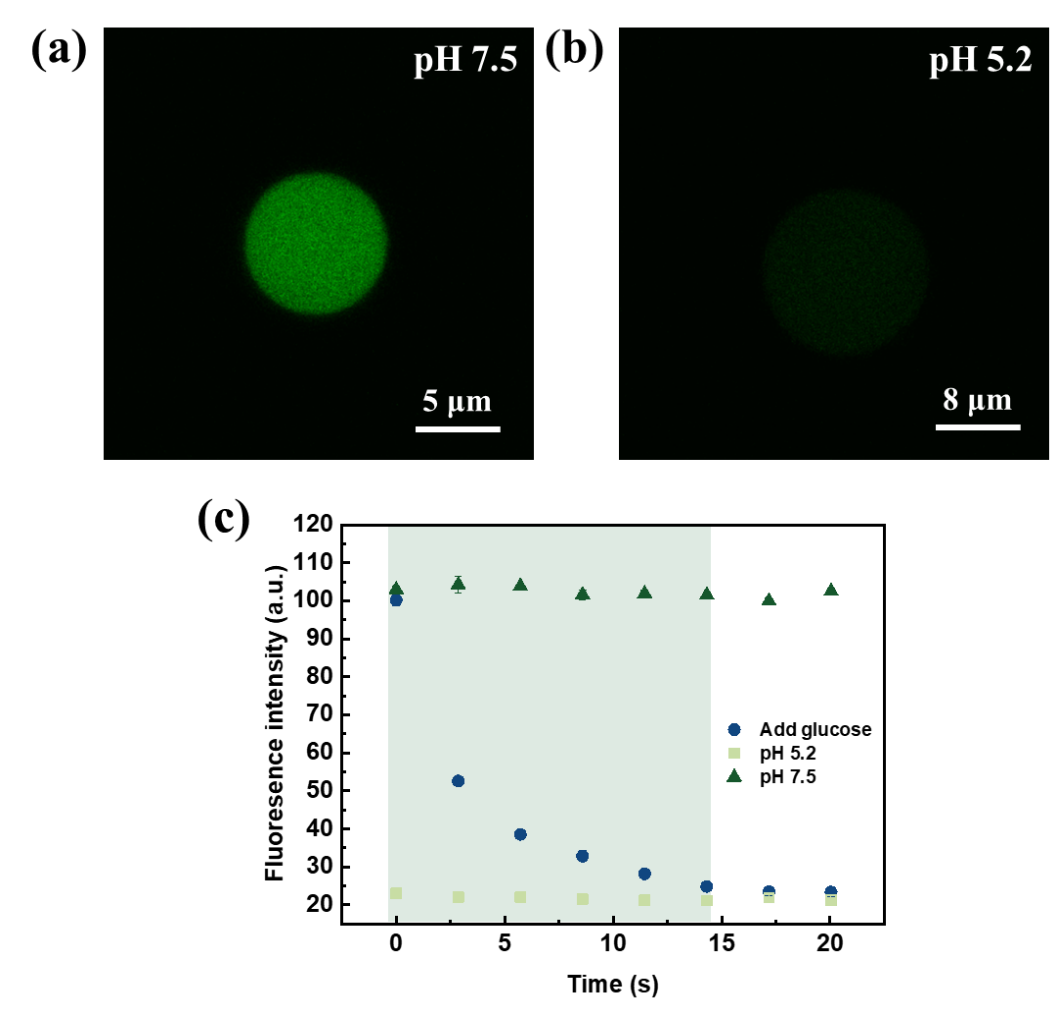


**Figure S20.** (a, b) The fluorescence intensity of FITC in Coa@DMPC, loaded with GOx, at different pH values. (c) Fluorescence changes of FITC inside Coa@DMPC loaded with GOx by adding glucose (glucose (1.5 mg/mL)).

It is observed that the fluorescence intensity of FITC at pH 5.2 is lower than that at pH 7.5 (a, b). As the pH decreases in (c), the fluorescence intensity of FITC decreases to a level of pH 5.2 very fast.

**Figure S21.** Study on the docking stability of RhB-Azo-Psomes (15 wt%, red) on FITC-Coa@DMPC surface (green) after pH reduction to about pH 6.5 for 70 h. (
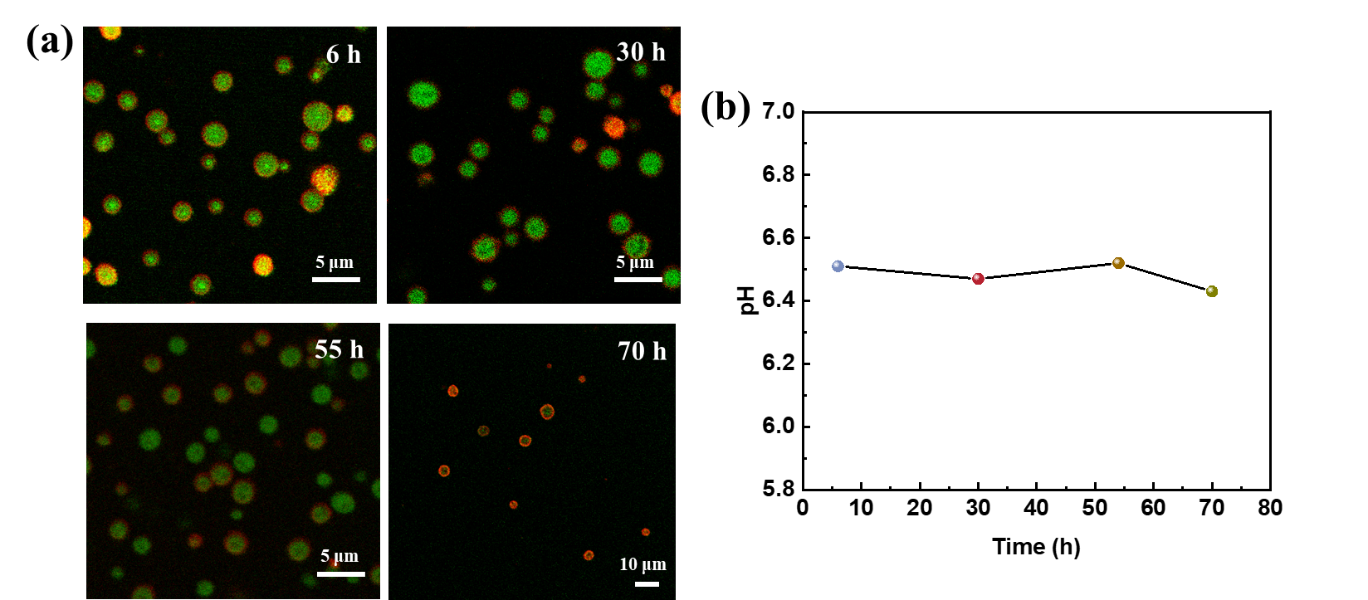
a) CLSM images of the binding of RhB-Azo-Psomes to FITC-Coa@DMPC after pH reduction at different time points. (b) The pH of the corresponding HMC solution at different time points.

During the first 55 hours, HMC maintain high stability at an environmental pH of 6.5. At 70 hours, due to the decreased stability of Coa@DMPC, large number of Coa@DMPC dissociates, as the Azo-Psomes on their surface are released into the environment and subsequently docked onto undissociated Coa@DMPC. Therefore, it is observed that the number of HMC decreases but the fluorescence of Azo-Psomes on the surface of the remaining HMC is enhanced.


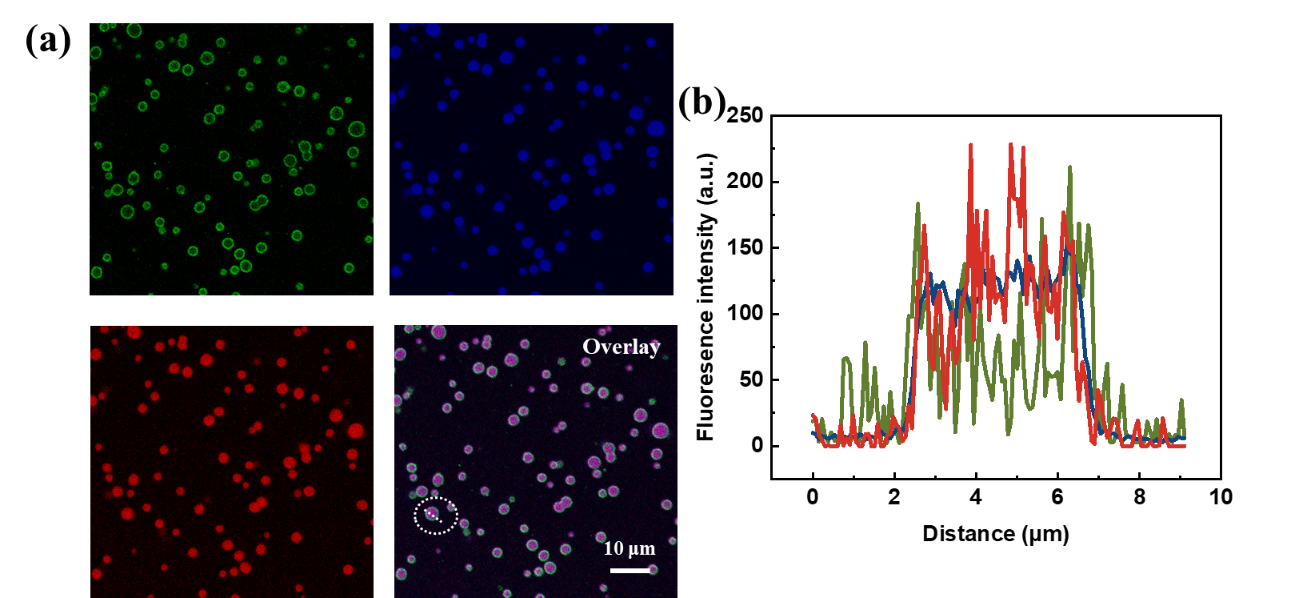


**Figure S22.** (a) CLSM images of enzyme-loaded Coa@DMPC as control experiment for **Figures 5b**-**c**, showing the preferentially assembled DMPC (NBD-PC, green) on the surface of Coa@DMPC that is loaded with Cy7-PAL (red) and Cy5-GOx (blue). (b) Corresponding fluorescence intensity analysis (position of imaginary line is shown in (a).

**Figure S23.** ^1^H NMR (500 MHz, CDCl_3_) of PDSMA-BCP A.

PDSMA-BCP A is used for the formation of Cy5-labeled Azo-Psomes. Further details are explained under points **3.7** and **3.9**.

**Figure S24.** The loading efficiency of L-Phenylalanine after optimization with Azo-Psomes (15 wt%) was 82%.

**Figure S25.** The pH at different time points after the release of L-phenylalanine by Azo-Psomes (15 wt%). Acidified solution (pH 6.5) of L-Phenylalanine-loaded Azo-Psomes by the addition of HCl to start the release of L-Phenylalanine and the immediate PAL conversion into NH_3_ to induce pH jump, finalized by the acidification through the addition of HCl (L-phenylalanine (1.5 mg/mL)).


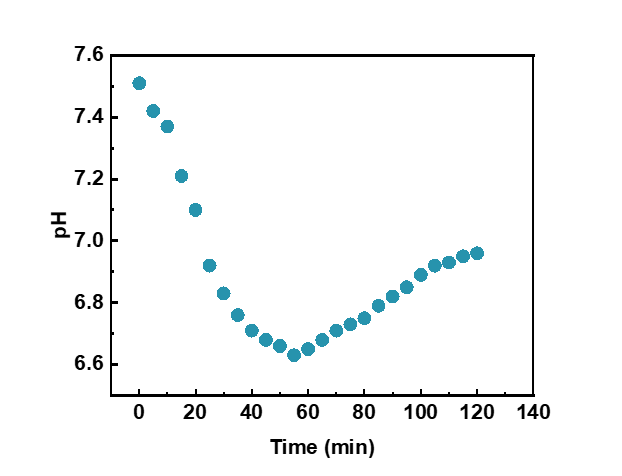


**Figure S26.** Potential of one-time release of L-phenylalanine within undocking system. Azo-Psomes loaded with L-phenylalanine (Initial concentration is 2.5 mg/mL) in the presence of coacervate droplets, loaded with GOx and PAL.

In the undocking system, increasing the amount of L-phenylalanine leads to more NH_3_ production. Therefore, the pH jump could over a longer reaction time, exhibiting a similar change process as in the docking system, using here a lower concentration of L-phenylalanine (**Figure 5d**).


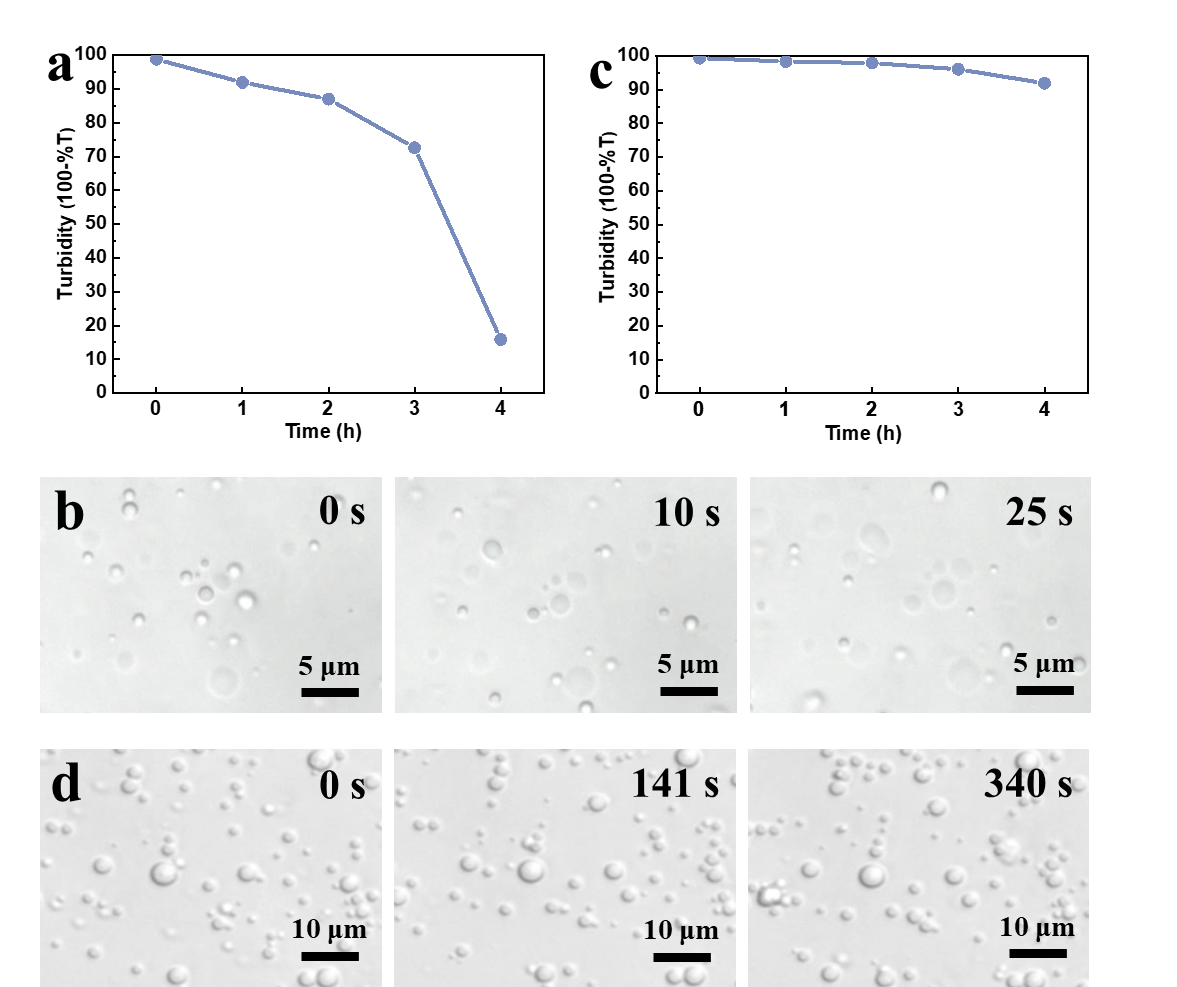


**Figure S27:** (a) Turbidity measurements of membrane-less coacervates at different time points. (b) Time-dependent corresponding bright-field images of membrane-less coacervates at different time points. (c) Turbidity measurements of Coa@DMPC at different time points. (d) Time-dependent corresponding bright-field images of Coa@DMPC at different time points.

Without the support of a phospholipid membrane, the stability of membrane-less coacervates is poor. This is proven by the linear decline of turbidity within 4 h (the lower turbidity means less coacervates in the solution). The dissociation process of the membrane-less coacervates is directly observed under the microscope. In contrast, Coa@DMPC is highly stable under the protection of a cholesterol-stabilized phospholipid membrane. There is a little turbidity change within 4h, leading to no dissociation under the microscope for a long time. These results further confirm and correlate with the discussed pH stability of Coa@DMPC (**Figure S28**) in the “Results and Discussion” part.


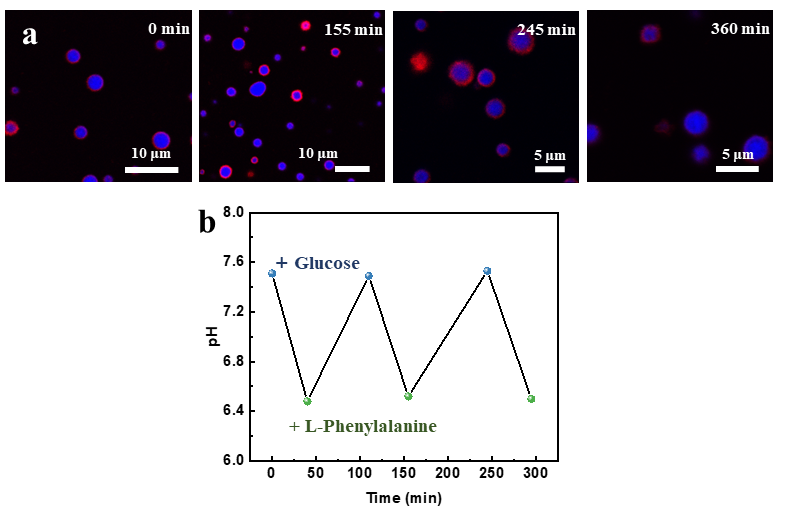


**Figure S28.** (a) CLSM images of HMC, showing the RhB-Azo-Psomes (15 wt%) bound to Coa@DMPC loaded with PAL and GOx (Cy5, blue) maintaining good docking of Azo-Psomes despite cyclic addition of glucose and L-Phenylalanine to mimic pH homeostasis. (b) Biomimetic pH homeostasis after cyclic addition of glucose and L-Phenylalanine HMC, visualized in (a) (L-phenylalanine (1.5 mg/mL), glucose (1.5 mg/mL)).

**Figure S29.** The loading efficiency of (β-CD)_2_Hemin after optimization with (β-CD)_2_Hemin-Psomes was 63%.

**Figure S30.** Results of asymmetric flow-field flow fractionation (AF4-LS) for Azo-Psomes (15 wt%) to determine molar mass (b), scaling parameter (b) and rho parameter (**Figure 2c**). a) Detector signals (solid line - LS, dashed line - RI, dotted line - UV) and radius of gyration (R_g_, symbol) of Azo-Psomes as a function of elution time obtained by AF4-LS. b) scaling plot of R_g_ (symbols) as function of molar mass determined by AF4-LS.

Briefly, the AF4-LS results for the investigated Azo-Psomes (15 wt%) outline that they possess the following properties (**Figures 2c**, **S30**): They exhibit higher values of ρ (R_g_/R_h_) parameter (≤ 1.2, **Figure 2c**) compared to theoretical values for hard spheres (ρ = 0.778) leading to the assumption that hollow spheres^[11]^ are formed in the molar mass range of 10^8^-10^9^ g/mol. This observation corresponds to previously published data for Azo-Psomes (25 and 50 wt%).^[7]^ Due to the assembly of two BCPs in Azo-Psomes (15 wt%), these may have a heterogeneous surface composition, with the Azo-BCP having a longer PEG tail. The determined scaling parameter (ν) (**Figure S30b**) with a value of 0.33 confirms the desired spherical morphology of Azo-Psomes (15wt%), too.^[11]^ Considering the batch DLS results, there is a good correlation between these and the AF4-LS results. More challenging is the interpretation of the conformation in the early-eluting, additional sample fraction with smaller sizes visible in the AF4-LS fractogram as a signal shoulder up to 19 min (**Figure S30a**) and with molar masses lower than 10^8^ g/mol (**Figure 30b**). A possible explanation for this behavior could be that irregularly assembled BCPs form smaller, asymmetric micelles with protruding longer chains that exhibit greater flexibility, which coexist in the Azo-Psomes (15 wt%) sample and are not removed during purification by hollow fiber filtration (using a MWCO of 1000 kDa). This could explain the larger ρ parameter below 10^8^ g/mol in **Figure 2c**.

# 5. References

[1] a) S. Moreno, H. Hubner, C. Effenberg, S. Boye, A. Ramuglia, D. Schmitt, B. Voit, I. M. Weidinger, M. Gallei, D. Appelhans, *Biomacromolecules* **2022**, *23*, 4655-4667; b) H. Gumz, S. Boye, B. Iyisan, V. Kronert, P. Formanek, B. Voit, A. Lederer, D. Appelhans, *Adv Sci (Weinh)* **2019**, *6*, 1801299.

[2] J. Li, X. Liu, L. Abdelmohsen, D. S. Williams, X. Huang, *Small* **2019**, *15*, 1902893.

[3] S. Wang, Z. Xu, S. Lin, X. Liu, L. Wang, X. Huang, *Science China Technological Sciences* **2020**, *63*, 1416-1425.

[4] J. L. Pinol R, G. F, L. D, A. PA, K. P, C. A, L. M.-H., *Macromolecules* **2007**, *40*, 5625-5627.

[5] a) C. Zhao, M. Zhu, Y. Fang, X. Liu, L. Wang, D. Chen, X. Huang, *Mater. Horiz.* **2020**, *7*, 157-163; b) L. Jia, D. Cui, J. Bignon, A. Di Cicco, J. Wdzieczak-Bakala, J. Liu, M. H. Li, *Biomacromolecules* **2014**, *15*, 2206-2217.

[6] K. Zhang, S. Moreno, X. Wang, Y. Zhou, S. Boye, D. Voigt, B. Voit, D. Appelhans, *Biomacromolecules* **2023**, *24*, 2489-2500.

[7] K. Zhang, Y. Zhou, S. Moreno, S. Schwarz, S. Boye, B. Voit, D. Appelhans, *J Colloid Interface Sci* **2024**, *654*, 1469-1482.

[8] A. Dreßen, T. Hilberath, U. Mackfeld, A. Billmeier, J. Rudat, M. Pohl, *Journal of Biotechnology* **2017**, *258*, 148-157.

[9] F. W. Studier, *Protein Expression and Purification* **2005**, *41*, 207-234.

[10] S. Fritzsche, F. Tischer, W. Peukert, K. Castiglione, *Reaction Chemistry & Engineering* **2023**, *8*, 2156-2169.

[11] A. Lederer, W. Burchard, *Hyperbranched polymers: Macromolecules in between deterministic linear chains and dendrimer structures*, **2015**.
